# Engineering a *Pseudomonas taiwanensis* 4-coumarate platform for production of *para*-hydroxy aromatics with high yield and specificity

**DOI:** 10.1016/j.ymben.2023.05.004

**Published:** 2023-07

**Authors:** Benedikt Wynands, Franziska Kofler, Anka Sieberichs, Nadine da Silva, Nick Wierckx

**Affiliations:** Institute of Bio- and Geosciences, IBG-1: Biotechnology, Forschungszentrum Jülich GmbH, 52425, Jülich, Germany

**Keywords:** Aromatics, Biocatalysis, 4-Coumarate, Metabolic engineering, Phenylalanine/tyrosine ammonia-lyase, *Pseudomonas*

## Abstract

Aromatics are valuable bulk or fine chemicals with a myriad of important applications. Currently, their vast majority is produced from petroleum associated with many negative aspects. The bio-based synthesis of aromatics contributes to the much-required shift towards a sustainable economy. To this end, microbial whole-cell catalysis is a promising strategy allowing the valorization of abundant feedstocks derived from biomass to yield *de novo*-synthesized aromatics.

Here, we engineered tyrosine-overproducing derivatives of the streamlined *chassis* strain *Pseudomonas taiwanensis* GRC3 for efficient and specific production of 4-coumarate and derived aromatics. This required pathway optimization to avoid the accumulation of tyrosine or *trans*-cinnamate as byproducts. Although application of tyrosine-specific ammonia-lyases prevented the formation of *trans*-cinnamate, they did not completely convert tyrosine to 4-coumarate, thereby displaying a significant bottleneck. The use of a fast but unspecific phenylalanine/tyrosine ammonia-lyase from *Rhodosporidium toruloides* (*Rt*PAL) alleviated this bottleneck, but caused phenylalanine conversion to *trans*-cinnamate. This byproduct formation was greatly reduced through the reverse engineering of a point mutation in prephenate dehydratase domain-encoding *pheA*. This upstream pathway engineering enabled efficient 4-coumarate production with a specificity of >95% despite using an unspecific ammonia-lyase, without creating an auxotrophy. In shake flask batch cultivations, 4-coumarate yields of up to 21.5% (Cmol/Cmol) from glucose and 32.4% (Cmol/Cmol) from glycerol were achieved. Additionally, the product spectrum was diversified by extending the 4-coumarate biosynthetic pathway to enable the production of 4-vinylphenol, 4-hydroxyphenylacetate, and 4-hydroxybenzoate with yields of 32.0, 23.0, and 34.8% (Cmol/Cmol) from glycerol, respectively.

## Highlights

• Screening of multiple TALs and a PAL/TAL in *Pseudomonas* for 4-coumarate production.
• Upstream pathway engineering to reduce byproduct formation when applying PAL/TAL.
• PheA^P144S^ reduces flux to phenylalanine without causing auxotrophy.
• 4-Coumarate production from glucose and glycerol with high yield and specificity.
• Product diversification to 4-vinylphenol, 4-hydroxyphenylacetate, 4-hydroxybenzoate.

## Graphical abstract

**Figure:**
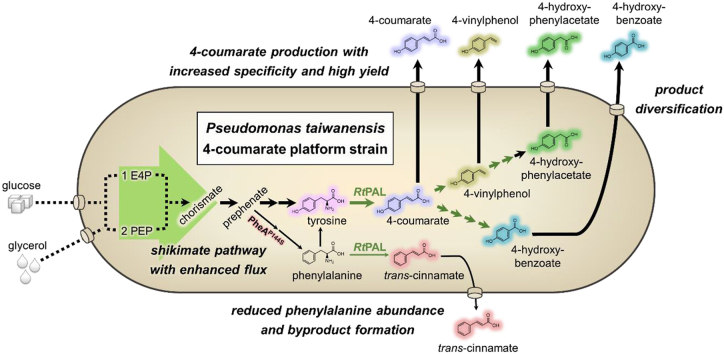

## 1 Introduction

The bio-based production of valuable chemicals, including aromatics, is an essential requirement to gain independence from dwindling fossil resources and polluting petrochemical processes that currently serve as the primary source for the synthesis of many of such chemicals. Aromatic compounds have an exceptionally high industrial relevance due to their large production volume and versatile application range, including their use as building blocks for polymers or nutra- and pharmaceuticals (Huccetogullari et al., 2019; Noda and Kondo, 2017).

Microbial catalysis is a promising approach to produce bulk and fine chemicals from inexpensive renewable feedstocks in eco-friendly processes, thereby contributing to the much-required transition towards a sustainable bioeconomy (Intasian et al., 2021; Lee et al., 2019). In this context, microbial production strains should be designed “with the end in mind” (Straathof et al., 2019), minimizing potential hurdles in subsequent scale-up of the production process. This entails an efficient production in terms of titer, yield, and rate, but also other factors such as ease of cultivation, genetic stability, avoidance of the use of antibiotics, and high product specificity without byproducts (Blombach et al., 2022). Pseudomonads have been extensively developed as hosts for the bioproduction of a wide range of value-added chemicals (Loeschcke and Thies, 2020; Nikel and de Lorenzo, 2018). The ease of cultivation and their inherent resilience towards toxicants (Bitzenhofer et al., 2021; Kusumawardhani et al., 2018) are crucial benefits rendering them high-potential *chassis* for aromatics biocatalysis (Schwanemann et al., 2020; Tiso et al., 2014). A wealth of available synthetic biology tools make *Pseudomonas* readily amenable to stable genomic engineering (Martin-Pascual et al., 2021).

Previously, we applied solvent-tolerant *Pseudomonas taiwanensis* VLB120 and its streamlined genome-reduced *chassis* strains (Wynands et al., 2019) as microbial cell factories for *de novo* production of 4-hydroxybenzoate and phenol (Lenzen et al., 2019; Wynands et al., 2018, 2019). In this context, the metabolism of *P. taiwanensis* GRC3 was rewired to overproduce tyrosine (Wynands et al., 2019). Specifically, (hydro)aromatics degradation pathways were deleted (Δ*pobA*, Δ*hpd*, Δ*quiC*, Δ*quiC1*, Δ*quiC2* = Δ5) to prevent growth on 4-hydroxybenzoate, tyrosine, and quinate/shikimate, and thus product and/or precursor loss. Furthermore, point mutations were implemented in *trpE* (P290S), *aroF-1* (P148L), and *pheA* (T310I) to increase the flux into the shikimate pathway. The mutation in *trpE* limits tryptophan biosynthesis and likely alleviates allosteric inhibition of enzymes and repression of genes involved in the shikimate pathway (Wierckx et al., 2008; Wynands et al., 2018). The modifications of AroF-1 (DAHP synthase) and PheA (bifunctional chorismate mutase/prephenate dehydratase) cause feedback-inhibition resistance (Weaver and Herrmann, 1990; Zhang et al., 1998), thereby debottlenecking the respective catalyzed rate-limiting reactions. Finally, pyruvate kinase A was deleted (Δ*pykA*) to enhance the phosphoenolpyruvate precursor supply. The resulting strain, *P. taiwanensis* GRC3Δ5-*trpE*^P290S^-*aroF-1*^P148L^*-pheA*^T310I^Δ*pykA* (here denoted as GRC3Δ5-TYR2) (Wynands et al., 2019), is an ideal platform for the bioproduction of tyrosine-derivable aromatics. Moreover, this strain was adapted to produce phenylalanine-derived aromatics by deleting genes encoding phenylalanine hydroxylase (Δ*phhAB*) and other enzymes involved in phenylalanine catabolism (Δ*katG*, ΔPVLB_10925), enabling the production of *inter alia trans*-cinnamate (Otto et al., 2019) and benzoate (Otto et al., 2020) upon product pathway implementation.

**Fig. 1:**
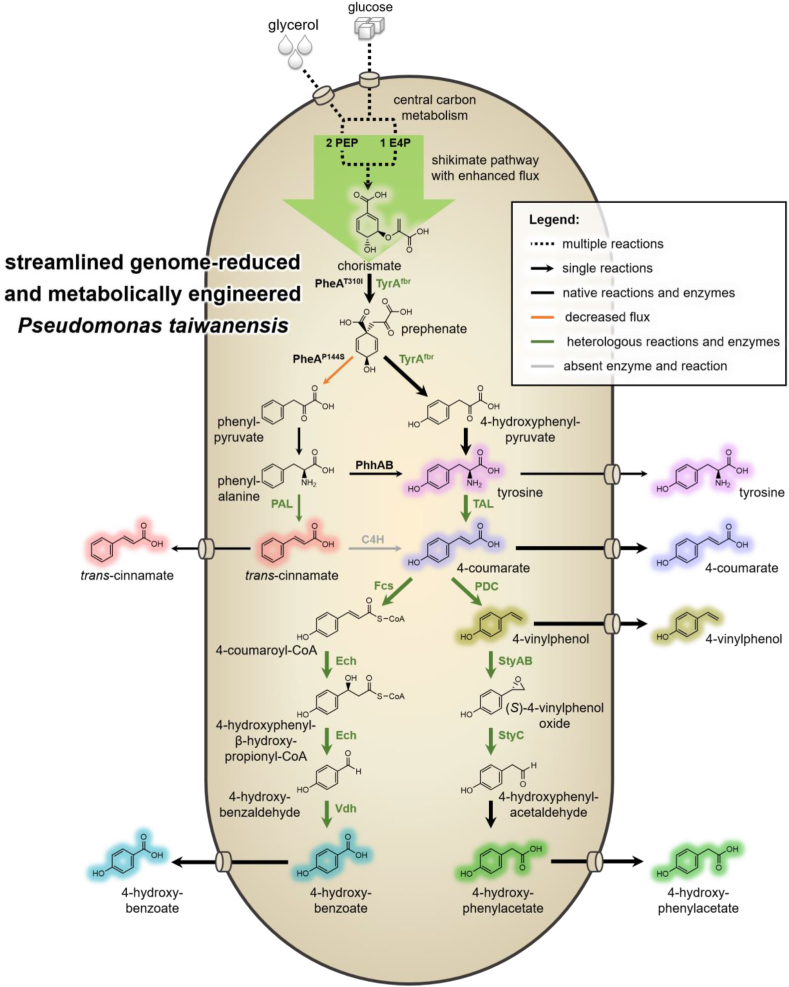
Biosynthetic pathways for the production of 4-coumarate and derived aromatics. Heterologous enzymes and introduced reactions are indicated in green. The orange arrow indicates a decreased flux from prephenate to phenylpyruvate due to the PheA^P144S^ mutation. The grey arrow specifies the reaction catalyzed by plant P450 cinnamate 4-hydroxylase (not applied in this study). Dashed arrows represent multiple reactions. Abbreviations: PEP, phosphoenolpyruvate; E4P erythrose 4-phosphate.

In the here presented study, we focused on the production of 4-coumarate and aromatics derived thereof (Fig. 1). 4-Coumarate is a valuable compound with pharma- and nutraceutical applications, and it can serve as a building block for polymers (Timokhin et al., 2020). Additionally, it is a precursor of several other industrially relevant commodity aromatics, including 4-vinylphenol (Kang et al., 2015), 4-hydroxyphenylacetate (Shen et al., 2019), and 4-hydroxybenzoate (Yu et al., 2016), whose production was also demonstrated in this study. Valuable non-aromatic chemicals including *cis,cis*-muconate (Johnson et al., 2016) and β-ketoadipate (Fenster et al., 2022) that can be used for nylon production can also be derived from 4-coumarate. However, 4-coumarate can also serve as starting unit for the synthesis of high-value secondary metabolites such as flavonoids and stilbenoids (Milke et al., 2018).

Natural biosynthesis of 4-coumarate relies either on the enzymatic deamination of phenylalanine followed by *para*-hydroxylation of *trans*-cinnamate, or on the deamination of tyrosine (Fig. 1). Both pathways have been applied in microbial cell factories to enable biotechnological 4-coumarate production (Li et al., 2018; Liu et al., 2019; Vannelli et al., 2007; Vargas-Tah and Gosset, 2015). The hydroxylation of *trans*-cinnamate to 4-coumarate, however, depends on plant cytochrome P450 cinnamate 4-hydroxylase (C4H), whose functional expression in bacteria is challenging and has only resulted in low 4-coumarate production in *E. coli* (Li et al., 2018). Hence, this pathway was not applied in the here presented study and 4-coumarate production was solely based on the deamination of tyrosine. This reaction is catalyzed either by tyrosine ammonia-lyases (TALs) that are substrate-specific or by phenylalanine/tyrosine ammonia-lyases (PAL/TALs) that also show activity towards phenylalanine. Although PAL/TALs generally have higher catalytic activity than TALs (Hendrikse et al., 2020), their *in vivo* application can cause the formation of *trans-*cinnamate as a byproduct. This is particularly relevant in *Pseudomonas*, because unlike other biotechnological hosts this microbe anabolizes considerable amounts of tyrosine from phenylalanine through *para*-hydroxylation catalyzed by PhhAB (Otto et al., 2019; Wierckx et al., 2008).

## 2 Results and discussion

### 2.1 Establishing production of 4-coumarate through tyrosine deamination and upstream pathway engineering

Specific deamination of tyrosine is a crucial prerequisite to prevent accumulation of *trans*-cinnamate, the deamination product of phenylalanine, as a byproduct. Ammonia-lyases with a high tyrosine specificity, however, often suffer from lower catalytic efficiencies compared to aromatic amino acid ammonia-lyases with a broader substrate spectrum (Hendrikse et al., 2020). Due to different conditions, *in vitro* activities cannot easily be translated into the *in vivo* performance of tyrosine-deaminating ammonia-lyases that additionally can severely differ between microbial hosts (Jendresen et al., 2015). Consequently, a screening of different ammonia-lyases within the host strain is required.

**Fig. 2:**
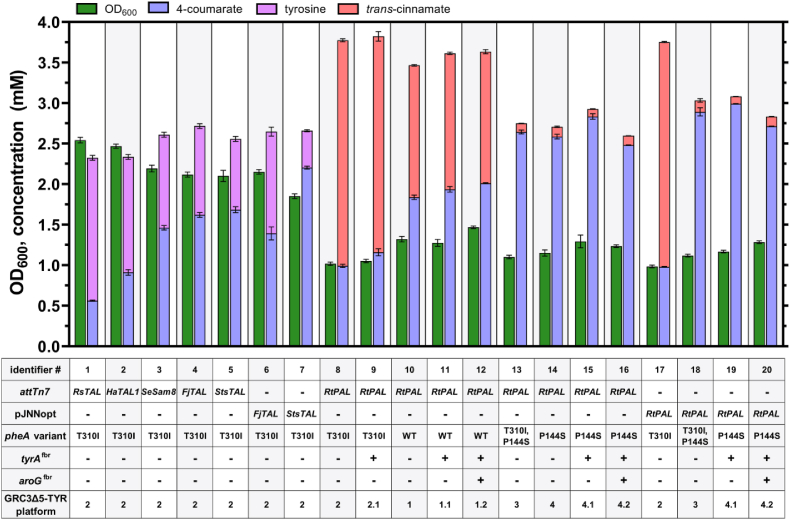
4-Coumarate production using ammonia-lyases from *R. sphaeroides* (*Rs*TAL), *H. aurantiacus* (*Ha*TAL1), *S. espanaensis* (*Se*Sam8), *F. johnsoniae* (*Fj*TAL), *Streptomyces* sp. (*Sts*TAL), and *R. toruloides* (*Rt*PAL). *PAL*/*TAL* genes were either chromosomally (*attTn7*) or episomally (pJNNopt) expressed in tyrosine-overproducing *P. taiwanensis* GRC3Δ5-TYR2. *Rt*PAL was also assessed in combination with different *pheA* genotypes and/or heterologous expression of *tyrA*^fbr^ and *aroG*^fbr^ that were integrated into the chromosome under the control of synthetic promoter *P*_*14e*_. Strains were grown in 24-well microtiter plates using mineral salt medium (MSM, three-fold-buffered) with 20 mM glucose as sole carbon source for 96 h to ensure glucose depletion. Error bars indicate the standard deviation of biological replicates. For selected strains, the cultivation experiment was performed multiple times (n ≥ 3).

In order to enable specific *de novo* production of 4-coumarate in the *P. taiwanensis* GRC3Δ5-TYR2 platform strain (Wynands et al., 2019) through tyrosine deamination, several tyrosine ammonia-lyases (TALs) were screened. Due to the absence of the ferulic acid degradation pathway in *P. taiwanensis* VLB120 (Lenzen et al., 2019), this strain – unlike many other Pseudomonads – is unable to metabolize ferulate and 4-coumarate (Fig. S1) and a deletion of the associated genes (*ech-vdh-fcs*) is not required to achieve 4-coumarate accumulation upon heterologous expression of a tyrosine-deaminating enzyme. TALs from *Rhodobacter sphaeroides* (*Rs*TAL), *Herpetosiphon aurantiacus* (*Ha*TAL1), *Saccharothrix espanaensis* (*Se*Sam8), *Flavobacterium johnsoniae* (*Fj*TAL) (Jendresen et al., 2015), and *Streptomyces* sp. NRRL F-4489 (*Sts*TAL) (Cui et al., 2020) were cloned into the pBG14f_*FRT_Kan* plasmid backbone (Ackermann et al., 2021) to allow targeted transpositional delivery into the chromosomal Tn7 attachment site (*attTn7*). A phenylalanine ammonia-lyase (PAL) from *Rhodosporidium toruloides* (*Rt*PAL) (Nijkamp et al., 2007) with PAL/TAL activity was also inserted as a control. Gene expression was driven by the strong constitutive synthetic promoter *P*_*14f*_ (Zobel et al., 2015). Production was tested in 24-well microtiter plates with mineral salt medium (MSM) containing 20 mM glucose (Fig. 2).

Due to the high specificity of the tested TALs, 4-coumarate was produced without considerable formation of *trans*-cinnamate (<0.002 mM) (Fig. 2). However, none of these TALs (#1 to #5) enabled a sufficient conversion rate, which is apparent by the detection of significant amounts of tyrosine (>0.8 mM) that remained unconverted. The best two TAL-expressing strains enabled production of 1.62 ± 0.07 (*Fj*TAL, #4) and 1.68 ± 0.09 mM (*Sts*TAL, #5) 4-coumarate with 1.10 ± 0.07 (*Fj*TAL, #4) and 0.87 ± 0.08 mM (*Sts*TAL, #5) residual tyrosine. Of note, *Fj*TAL and *Sts*TAL were codon-optimized for *P. taiwanensis* VLB120, unlike the others that were codon-optimized for *E. coli* (Jendresen et al., 2015), indicating that a potentially enhanced translation could be impacting the strains’ performances. The bottleneck of tyrosine deamination remained even with strong overexpression of *Fj*TAL and *Sts*TAL using pJNNopt-derived plasmids (Neves et al., 2019). For GRC3Δ5-TYR2 pJNNopt-*StsTAL* (#7), tyrosine formation was only moderately decreased to 0.46 ± 0.02 mM, while 4-coumarate production was even negatively affected with episomally expressed *Fj*TAL (#6).

Contrarily, GRC3Δ5-TYR2 with chromosomally integrated *Rt*PAL (*attTn7*::*FRT_Kan_P*_*14f*_*-RtPAL*, #8) showed no residual tyrosine (Fig. 2). However, due to the enzyme's low substrate specificity, phenylalanine was deaminated to *trans*-cinnamate as a major byproduct whose titer (2.78 ± 0.04 mM) even surpassed that of 4-coumarate (0.99 ± 0.05 mM). In *Pseudomonas*, a substantial proportion of anabolized tyrosine results from *para*-hydroxylation of phenylalanine (Otto et al., 2019; Wierckx et al., 2008) and the implemented *pheA*^T310I^ modification likely further increases the flux from prephenate to phenylalanine due to an alleviated allosteric inhibition of the encoded bifunctional chorismate mutase/prephenate dehydratase and metabolic channeling. Therefore, an identical strain without the *pheA*^T310I^ mutation, GRC3Δ5-TYR1, chromosomally expressing *Rt*PAL, was tested. Indeed, this strain (#10) produced more 4-coumarate (1.84 ± 0.06 mM) and less *trans*-cinnamate (1.63 ± 0.02 mM) (Fig. 2), although the total phenylpropanoid production (i.e., 4-coumarate + *trans*-cinnamate) was also somewhat reduced. In order to further enhance the flux into the tyrosine biosynthesis branch, codon-optimized *tyrA*^fbr^ – encoding feedback inhibition-resistant bifunctional chorismate/prephenate dehydrogenase from *E. coli* – was integrated into the intergenic region of PVLB_23545/40 that was previously characterized as a suitable landing pad for strong heterologous gene expression (Köbbing et al., manuscript in preparation). Despite causing a negative effect on growth in tyrosine producers (Fig. S2), constitutive overexpression of *tyrA*^fbr^ using the synthetic promoter *P*_*14e*_ (Zobel et al., 2015) only showed a minor – yet significant – increase regarding 4-coumarate production from 0.99 ± 0.05 to 1.16 ± 0.09 mM in the PheA^T310I^ background (#8 vs. #9) and 1.84 ± 0.06 to 1.94 ± 0.08 mM with wild-type PheA (#10 vs. #11). The accumulation of high *trans*-cinnamate concentrations (2.66 ± 0.12 mM for #9 and 1.68 ± 0.04 mM for #11) could still not be prevented (Fig. 2). In theory, deleting the prephenate dehydratase domain of *pheA* would eliminate the flux from prephenate towards phenylalanine and *trans*-cinnamate entirely, but this would also cause a phenylalanine auxotrophy and thus the need for costly supplementation of this aromatic amino acid. Additionally, the deletion of *pheA* caused a severe growth defect in *Pseudomonas putida* even in rich medium (Kuepper et al., 2015; Yu et al., 2016). In Kuepper et al. (2015), the growth of a Δ*pheA* mutation-harboring *P. putida* strain in minimal medium could only be fully restored with supplementation of high phenylpyruvate concentrations (>5 mM), and this supplemented phenylalanine precursor would likely still be converted to *trans*-cinnamate. A conditionally auxotrophic strain with a dynamic flux towards tryptophan was recently reported for anthranilate-producing *P. putida* (Fernández-Cabezón et al., 2022). This elegant approach could be interesting to limit phenylalanine and thus *trans*-cinnamate formation in a cell density-dependent manner. However, we observed growth-coupled production of 4-coumarate and relied on constitutive *Rt*PAL expression. Therefore, we wanted to assess a reduced prephenate dehydratase activity to decrease the abundance of phenylalanine as an *Rt*PAL substrate on the level of enzyme activity rather than on the level of enzyme expression. Previously Nijkamp et al. (2007) obtained an *Rt*PAL-expressing 4-coumarate-overproducing *P. putida* S12 strain that was generated through random mutagenesis, antimetabolite selection, and high-throughput screening. The respective strain featured a phenylalanine-bradytrophic phenotype indicating an altered prephenate dehydratase activity. As the genome sequence of this mutagenized strain, *P. putida* S12 C3, was not available, *pheA* was PCR-amplified from genomic DNA and analyzed by Sanger sequencing. Indeed, a mutation was identified resulting in an amino acid substitution in the prephenate dehydratase domain of PheA (P144S; CCG→TCG). A mutation causing the same amino acid substitution (P144S; CCG→AGC) was introduced in *pheA* of GRC3Δ5-TYR1 and GRC3Δ5-TYR2 to yield GRC3Δ5-TYR3 (with *pheA*^T310I,P144S^) and GRC3Δ5-TYR4 (with *pheA*^P144S^), respectively. Upon integration of *Rt*PAL into the Tn7 site, the respective strains (#13 and #14) produced only low amounts of *trans*-cinnamate (∼0.11 mM) and highly increased 4-coumarate titers of 2.64 ± 0.06 (#13) and 2.59 ± 0.07 mM (#14), respectively, with no detectable tyrosine remaining (Fig. 2). Thus, the PheA^P144S^ amino acid substitution was proven to be effective in limiting *trans*-cinnamate formation resulting in a highly increased specificity of 4-coumarate production. However, the overall phenylpropanoid production was significantly lower (2.75 ± 0.05 mM) for GRC3Δ5-TYR3-*attTn7*::*P*_*14f*_*-RtPAL* (#13) compared to that of the predecessor (#8) lacking the *pheA*^P144S^ mutation (3.77 ± 0.02 mM), indicating a detrimental effect of this modification on the production yield. This mutation also led to a decreased tyrosine formation in strains lacking *Rt*PAL (Fig. S4). The reduced tyrosine formation was concomitant with the appearance of several unidentified peaks in the HPLC chromatogram, indicating the generation of bottlenecks within the biosynthetic pathway (Fig. S5). Potentially, regulatory effects related to a reduced phenylalanine abundance could be causing this phenotype. However, phenylalanine-induced gene expression is mainly associated with catabolic pathways rather than anabolism (Herrera et al., 2010), and phenylalanine-limited growth could be expected to alleviate allosteric inhibition and cause derepression of the biosynthesis pathway. Therefore, the underlying mechanisms remain concealed. In addition to the reduced tyrosine formation, the introduction of *pheA*^P144S^ caused a severe effect on growth, negatively influencing proliferation, as shown for the tyrosine-producing platform strains in Fig. S3. This phenotype is likely directly linked to a reduced phenylalanine availability as the growth impairment was at least partially relieved when 0.1 mM phenylalanine was supplemented to the medium (Fig. S3).

4-Coumarate production was further enhanced by additional heterologous expression of *tyrA*^fbr^ in strains with *pheA*^P144S^ (2.83 ± 0.09 mM, #15), episomal expression of *Rt*PAL using pJNNopt-*RtPAL* (2.89 ± 0.09 mM, #18), and the combination of both (2.99 ± 0.01 mM, #19). Expression of *tyrA*^fbr^ in a *pheA*^T310I,P144S^ background resulted in extremely poor growth and irreproducible production (data not shown). Co-expression of codon-optimized feedback inhibition-resistant DAHP synthase (*aroG*^fbr^) in a cistron with *tyrA*^fbr^ did not significantly enhance (#11 vs. #12) or even significantly decreased production (#15 vs. #16 and #19 vs. #20).

### 2.2 4-Coumarate production profiles on glucose and glycerol

**Fig. 3:**
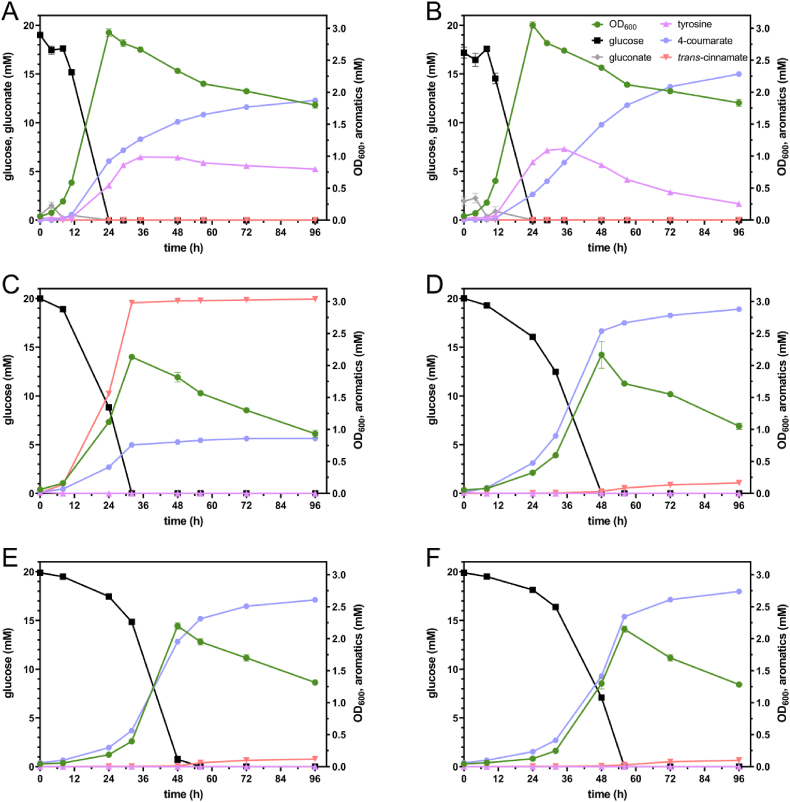
Shake flask cultivations of selected strains for the production of 4-coumarate: GRC3Δ5-TYR2 with pJNNopt-*FjTAL* (A), pJNNopt-*StsTAL* (B), or pJNNopt-*RtPAL* (C), GRC3Δ5-TYR3 with pJNNopt-*RtPAL* (D), GRC3Δ5-TYR3-*attTn7*::*P*_*14f*_-*RtPAL* (E), and GRC3Δ5-TYR4.1-*attTn7*::*P*_*14f*_-*RtPAL* (F) in MSM (two-fold-buffered) with 20 mM glucose. Error bars indicate the standard deviation of replicates (n = 3).

For a more detailed characterization of growth and production, selected strains were profiled over time in shake flasks (Fig. 3). The strains’ respective key performance indicators are shown in Table 1. GRC3Δ5-TYR2 with pJNNopt-*FjTAL* or pJNNopt-*StsTAL* showed relatively fast growth in MSM with 20 mM glucose reaching their maximum OD_600_ after 24 h (Fig. 3A and B). At that time, the carbon source was completely consumed and considerable accumulation of tyrosine and only relatively low 4-coumarate concentrations were observed. Tyrosine deamination slowly continued during the stationary phase until the end of cultivation, but displayed the major bottleneck. The slow conversion rate might result from transport limitations, or from product inhibition that has been reported for aromatic amino acid ammonia-lyases (Brack et al., 2022; Sariaslani, 2007). Although the final titer was lower for GRC3Δ5-TYR2 pJNNopt-*FjTAL* compared to GRC3Δ5-TYR2 pJNNopt-*StsTAL*, this strain showed a higher 4-coumarate production in the first 24 h (0.92 ± 0.01 vs. only 0.40 ± 0.00 mM). After 96 h, GRC3Δ5-TYR2 pJNNopt-*StsTAL* reached a 4-coumarate titer of 2.28 ± 0.02 mM with 0.26 ± 0.01 mM residual tyrosine (Fig. 3B), while GRC3Δ5-TYR2 pJNNopt-*FjTAL* showed production of only 1.88 ± 0.02 mM 4-coumarate with 0.80 ± 0.01 mM tyrosine (Fig. 3A). The overall higher combined production of 4-coumarate and tyrosine (2.67 ± 0.00 mM) of *Fj*TAL compared to that of *Sts*TAL (2.54 ± 0.01 mM) could be a result of the faster initial conversion rate of *Fj*TAL.

**Table 1:** Overview of 4-coumarate production performances.

Product	Strain	Carbon source	Growth rate (h^−1^)	Max. OD_600_	Product titer (mM)	Space-time yield (mM h^−1^)	Yield (%)^a^	Product specificity (%)^b^	Fig.
4-coumarate	GRC3Δ5-TYR2 pJNNopt-*FjTAL*	glucose	0.23 ± 0.01 (4–11 h)	2.93 ± 0.06 (24 h)	1.88 ± 0.02 (96 h)	0.020 ± 0.000 (0–96 h)	14.4 ± 0.1 (96 h)	70.1 ± 0.5 (96 h)	3A
	GRC3Δ5-TYR2 pJNNopt-*StsTAL*	glucose	0.25 ± 0.01 (4–11 h)	3.05 ± 0.05 (24 h)	2.28 ± 0.02 (96 h)	0.024 ± 0.000 (0–96 h)	17.9 ± 0.2 (96 h)	89.9 ± 0.6 (96 h)	3B
	GRC3Δ5-TYR2 pJNNopt-*RtPAL*	glucose	0.11 ± 0.00 (8–32 h)	2.13 ± 0.03 (32 h)	0.86 ± 0.02 (96 h)	0.009 ± 0.000 (0–96 h)	6.3 ± 0.2 (96 h)	22.1 ± 0.5 (96 h)	3C
	GRC3Δ5-TYR3 pJNNopt-*RtPAL*	glucose	0.08 ± 0.00 (8–48 h)	2.17 ± 0.21 (48 h)	2.88 ± 0.02 (96 h)	0.030 ± 0.000 (0–96 h)	21.5 ± 0.1 (96 h)	94.7 ± 0.1 (96 h)	3D
	GRC3Δ5-TYR3-*attTn7*::*P*_*14f*_*-RtPAL*	glucose	0.09 ± 0.00 (8–48 h)	2.20 ± 0.05 (48 h)	2.61 ± 0.01 (96 h)	0.027 ± 0.000 (0–96 h)	19.2 ± 0.0 (96 h)	95.7 ± 0.0 (96 h)	3E
	GRC3Δ5-TYR4.1-*attTn7*:: *P*_*14f*_*-RtPAL*	glucose	0.09 ± 0.00 (24–56 h)	2.15 ± 0.05 (56 h)	2.74 ± 0.01 (96 h)	0.028 ± 0.000 (0–96 h)	20.2 ± 0.0 (96 h)	96.5 ± 0.0 (96 h)	3F
	GRC3Δ5-TYR2 pJNNopt-*RtPAL*	glycerol	0.07 ± 0.00 (24–56 h)	2.15 ± 0.05 (72 h)	1.39 ± 0.01 (120 h)	0.011 ± 0.000 (0–120 h)	10.0 ± 0.1 (120 h)	26.4 ± 0.0 (120 h)	4A
	GRC3Δ5-TYR3 pJNNopt-*RtPAL*	glycerol	0.08 ± 0.00 (24–56 h)	2.75 ± 0.00 (72 h)	3.84 ± 0.01 (120 h)	0.032 ± 0.000 (0–120 h)	28.2 ± 0.1 (120 h)	95.7 ± 0.1 (120 h)	4B
	GRC3Δ5-TYR3-*attTn7*:: *P*_*14f*_*-RtPAL*	glycerol	0.07 ± 0.00 (24–56 h)	2.25 ± 0.02 (72 h)	4.51 ± 0.03 (144 h)	0.031 ± 0.000 (0–144 h)	32.4 ± 0.3 (144 h)	94.9 ± 0.1 (144 h)	4C

a “%” refers to carbon-molar percentage (% (Cmol/Cmol)).

b Product specificity refers to the 4-coumarate titer's proportion of the sum of quantified aromatics, i.e., 4-coumarate, tyrosine, and *trans*-cinnamate.

The maximum OD_600_ of GRC3Δ5-TYR2 pJNNopt-*RtPAL* was lower and reached later (2.13 ± 0.03 after 32 h) compared to those of the TAL-expressing strains (>2.9 after 24 h). This could be due to the higher toxicity resulting from increased phenylpropanoid concentrations (Fig. 3C) and a higher drain on phenylalanine and tyrosine as building blocks for protein biosynthesis. The identical strain additionally harboring the *pheA*^P144S^ modification, GRC3Δ5-TYR3 pJNNopt-*RtPAL*, reached its maximum OD_600_ even later (2.17 ± 0.21 after 48 h) and produced 2.88 ± 0.02 mM 4-coumarate and 0.16 ± 0.00 mM *trans*-cinnamate after 96 h (Fig. 3D). Noticeably, the cultures of GRC3Δ5-TYR3 pJNNopt-*RtPAL* turned brown and showed small dark aggregates at the end of cultivation (Fig. S6B). The browning could be associated to a yet unidentified catecholic byproduct that polymerizes upon autoxidation (Slikboer et al., 2015). However, this hypothesis remains to be investigated. Interestingly, this phenomenon was neither observed for GRC3Δ5-TYR2 pJNNopt-*RtPAL* (Fig. S6A) nor for the ancestor strain lacking the *Rt*PAL, but it did occur with other strains possessing the *pheA*^P144S^ mutation simultaneously expressing the *Rt*PAL (data not shown). Despite the impaired growth performance of GRC3Δ5-TYR3 pJNNopt-*RtPAL*, the maximum volumetric productivity was higher (0.102 ± 0.001 mM h^−1^ between 32 and 48 h) compared to GRC3Δ5-TYR2 expressing either *Fj*TAL (0.064 ± 0.001 mM h^−1^ between 11 and 24 h) or *Sts*TAL (0.045 ± 0.001 mM h^−1^ between 24 and 48h). Thus, GRC3Δ5-TYR3 pJNNopt-*RtPAL* featured the best 4-coumarate production rate in addition to the best titer and yield (Table 1). However, GRC3Δ5-TYR3-*attTn7*::*P*_*14f*_*-RtPAL* (Fig. 3E) behaved very similarly compared to the strain episomally expressing *Rt*PAL (Fig. 3D), and the final 4-coumarate titer was only slightly (but significantly lower) at 2.61 ± 0.01 mM. Given this minor difference, the chromosomal integration is considered to be advantageous over the application of plasmids due to an increased genetic stability and the avoidance of plasmid copy number variability (Jahn et al., 2014). Additionally, GRC3Δ5-TYR3-*attTn7*::*P*_*14f*_*-RtPAL* requires neither salicylate as an inducer for *Rt*PAL expression – due to the application of the constitutive *P*_*14f*_ promoter – nor antibiotics to ensure segregational plasmid retention, making it more suitable for scale-up.

GRC3Δ5-TYR4.1-*attTn7*::*P*_*14f*_*-RtPAL*, lacking the *pheA*^T310I^ modification but expressing heterologous *tyrA*^fbr^, produced significantly more 4-coumarate (2.74 ± 0.01 mM). However, the difference was minor, and the strain also suffered from a prolonged lag phase and thus delayed growth and production (Fig. 3F).

**Fig. 4:**
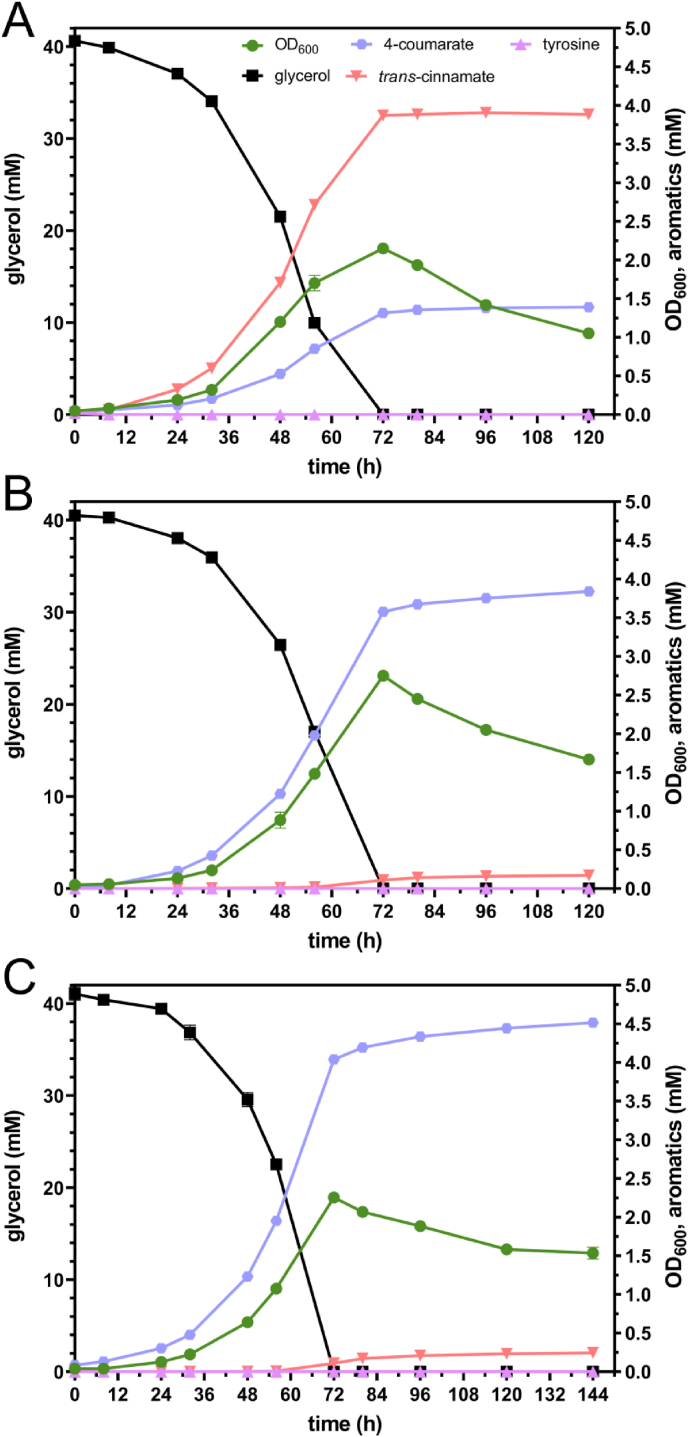
Shake flask cultivations of GRC3Δ5-TYR2 pJNNopt-*RtPAL* (A), GRC3Δ5-TYR3 pJNNopt-*RtPAL* (B), and GRC3Δ5-TYR3-*attTn7*::*P*_*14f*_-*RtPAL* (C) in MSM (two-fold-buffered) with 40 mM glycerol. Error bars indicate the standard deviation of replicates (n = 3).

In addition to glucose, also glycerol was used as sole carbon source in selected *Rt*PAL-expressing strains (Fig. 4). Glycerol is a major side stream of biodiesel production (Baskaran et al., 2021) and its valorization could add value to the biodiesel production process. Due to reduced growth rates on glycerol, all strains reached their maximum OD_600_ later than on glucose, after 72 h. GRC3Δ5-TYR2 pJNNopt-*RtPAL* produced 1.39 ± 0.01 mM 4-coumarate and 3.88 ± 0.01 mM *trans*-cinnamate, while GRC3Δ5-TYR3 pJNNopt-*RtPAL* reached a 4-coumarate titer of 3.84 ± 0.01 mM with only 0.17 ± 0.00 mM *trans*-cinnamate. The same strain expressing *Rt*PAL chromosomally, GRC3Δ5-TYR3-*attTn7*::*P*_*14f*_*-RtPAL*, even produced 4.51 ± 0.04 mM 4-coumarate with 0.24 ± 0.00 mM *trans*-cinnamate after 144 h. Thus, in contrast to the cultivation on glucose, on glycerol chromosomal expression of *Rt*PAL was better than episomal expression. This is likely due to the lower overall metabolic rates on glycerol, allowing lower expression of PAL that imposes less burden. For all strains, the higher titers translate into increased yields (Table 1) due to complete carbon source consumption. This is in line with our previous studies, in which we consistently achieved higher aromatics yields with glycerol (Lenzen et al., 2019; Otto et al., 2019, 2020; Wynands et al., 2018). This is likely related to a reduced flux through the Entner-Doudoroff pathway (Nikel et al., 2014) and consequently a higher impact of Δ*pykA* providing elevated phosphoenolpyruvate precursor supplies. In addition to that, strains harboring the *pheA*^P144S^ modification did not show brown color formation on glycerol while they did on glucose (Fig. S6D), again likely due to lower overall metabolic rates that alleviate side reactions at bottleneck points.

In summary, the here presented strategy to channel prephenate directly towards tyrosine, circumventing the phenylalanine loop by reducing the prephenate dehydratase activity through PheA^P144S^, allowed the application of an unspecific ammonia-lyase (PAL/TAL) to efficiently deaminate tyrosine to 4-coumarate with only minor formation of *trans*-cinnamate as byproduct. *Rt*PAL expression in combination with this upstream pathway engineering was superior for 4-coumarate production regarding all key process indicators (titer, rate, and yield) compared to the application of all tested tyrosine-specific ammonia-lyases despite the negative impact of *pheA*^P144S^ on growth. The upstream engineering strategy further enabled 4-coumarate production with much higher specificity (94.7–96.5%) compared to the use of TALs (70.7–89.9%), thereby facilitating downstream purification.

Efficient tyrosine conversion is a key determinant for high-yield 4-coumarate production because there is a distinct interplay between the platform strain and the applied ammonia-lyase whose activity is not only ultimately important for product formation but likely also indirectly affects production through an alleviated feedback inhibition or transcriptional derepression, resulting from the lower abundance of tyrosine. This is indicated by the fact that GRC3Δ5-TYR2 produced 2.03 ± 0.03 mM tyrosine from 20 mM glucose (Fig. S4), while the same strain episomally expressing *Sts*TAL or *Rt*PAL achieved higher aromatics production of 2.54 ± 0.01 mM (tyrosine + 4-coumarate, Figs. 3B) and 3.90 ± 0.02 mM (tyrosine + *trans*-cinnamate + 4-coumarate, Fig. 3C), respectively. Furthermore, GRC3Δ5-TYR3 (with *pheA*^P144S^) harboring pJNNopt-*Rt*PAL was able to outperform GRC3Δ5-TYR2-based producers expressing tyrosine-specific ammonia-lyases, even though GRC3Δ5-TYR3 showed a severely reduced tyrosine production (0.79 ± 0.00 mM) compared to GRC3Δ5-TYR2 (Fig. S4).

Overall, respectable carbon molar yields for 4-coumarate of up to 21.5 ± 0.1% on glucose (for GRC3Δ5-TYR3 pJNNopt-*RtPAL*) and 32.4 ± 0.3% on glycerol (for GRC3Δ5-TYR3-*attTn7*::*P*_*14f*_-*RtPAL*) were achieved. These yields are significantly higher than those reported for previous 4-coumarate-producing *P. putida* strains that were generated either rationally or non-rationally (Calero et al., 2016; Nijkamp et al., 2007).

### 2.3 Product diversification by biosynthetic pathway extension

One main advantage of the optimized 4-coumarate production platform is the fact that many value-added products can be derived from this metabolite, thereby reducing the time and effort required for engineering of individual production strain. To demonstrate this, the product spectrum was diversified by extending the biosynthetic pathway to obtain 4-vinylphenol, 4-hydroxyphenylacetate, and 4-hydroxybenzoate (Fig. 1). These compounds have versatile applications, including their use as building blocks for polymers and pharmaceuticals (Kang et al., 2015; Shen et al., 2019; Yu et al., 2016). Thus, their production is – like that of 4-coumarate – of industrial relevance, and a bio-based process is desired to gain independence from finite and polluting fossil resources. GRC3Δ5-TYR3 was used as a common platform strain to produce the target compounds upon product module integration. Required pathway genes were cloned into pBG14f_*FRT_Kan-RtPAL* downstream of *RtPAL* to allow chromosomal integration into *attTn7* and polycistronic co-expression.

**Fig. 5:**
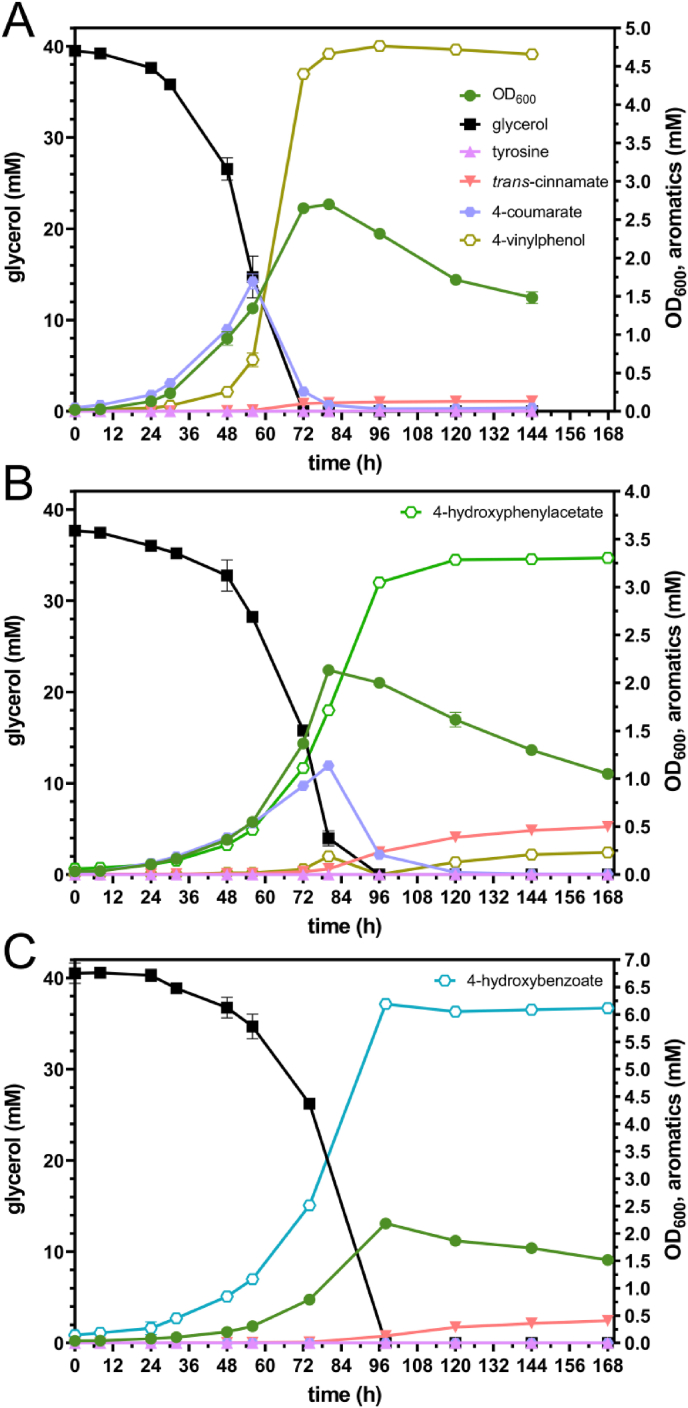
Shake flask cultivations of GRC3Δ5-TYR3 with integrated expression cassette of either *attTn7*::*Kan_FRT_P*_*14f*_-*RtPAL-pdc* (A), *attTn7*::*Kan_FRT_P*_*14f*_-*RtPAL-pdc-styABC* (B), or *attTn7*::*Kan_FRT_P*_*14f*_-*RtPAL-ech-vdh-fcs* (C) cultivated in MSM with 40 mM glycerol. The medium was one-fold-buffered for production of 4-vinylphenol and two-fold-buffered for production of 4-hydroxyphenylacetate and 4-hydroxybenzoate. Error bars indicate the standard deviation of replicates (n = 3).

For 4-vinylphenol, there is only one biosynthetic pathway available that relies on 4-coumarate decarboxylation. To enable its production, *RtPAL* was complemented with *para*-coumarate decarboxylase from *Lactobacillus plantarum* (*Lp*PDC), yielding strain GRC3Δ5-TYR3-*attTn7*::*P*_*14f*_-*RtPAL*-*pdc*. On 40 mM glycerol, this strain reached a maximum concentration of 4.77 ± 0.07 mM 4-vinylphenol (after 96 h) when glycerol was completely consumed, with transient formation of 4-coumarate which was completely decarboxylated at the end of the growth phase (Fig. 5A, Table 2). Strikingly, this is in the same range and even slightly higher than the titer achieved for 4-coumarate, despite 4-vinylphenol being more toxic to bacteria (Rodriguez et al., 2021) due to its higher hydrophobicity and its increased accumulation in the membrane causing its destabilization (Licandro-Seraut et al., 2013). Therefore, the higher titer indicates that product toxicity is not limiting production under these conditions. Potentially, the conversion of 4-coumarate reduced product inhibition of *Rt*PAL (Brack et al., 2022; Sariaslani, 2007). The transient accumulation of 1.69 ± 0.10 mM 4-coumarate after 56 h, however, indicates that 4-coumarate decarboxylation by *Lp*PDC is a bottleneck that potentially could be tuned to enhance production further in the future. Additionally, only 1.83 ± 0.03 mM 4-vinylphenol was produced from 20 mM glucose (Fig. S7B) and thus less than 4-coumarate (2.94 ± 0.13 mM) by an identical strain lacking *Lp*PDC (Fig. S7A). This indicates that a relieved product inhibition is likely not the cause for a higher 4-vinylphenol production on glycerol. The 4-vinylphenol yield of 32.0 ± 0.5% (Cmol/Cmol) from glycerol (Table 2) is considerably higher than the yields achieved in previous studies on microbial *de novo* production of 4-vinylphenol from glucose using *E. coli* (Gargatte et al., 2021; Kang et al., 2015; Qi et al., 2007), *P. putida* (Verhoef et al., 2009), or *Streptomyces* spp. (Fujiwara et al., 2016; Noda et al., 2015).

**Table 2:** Overview of production performances for 4-vinylphenol, 4-hydroxyphenylacetate, and 4-hydroxybenzoate.

Product	Strain	Carbon source	Growth rate (h^−1^)	Max. OD_600_	Product titer (mM)	Space-time yield (mM h^−1^)	Yield (%)^a^	Product specificity (%)^b^	Fig.
4-vinylphenol	GRC3Δ5-TYR3-*attTn7*:: *P*_*14f*_*-RtPAL-pdc*	glycerol	0.07 ± 0.00 (24–56 h)	2.70 ± 0.05 (80 h)	4.77 ± 0.00 (96 h)	0.049 ± 0.001 (0–96 h)	32.0 ± 0.5 (96 h)	96.8 ± 0.1 (96 h)	5A
4-hyrodxy-phenylacetate	GRC3Δ5-TYR3-*attTn7*:: *P*_*14f*_*-RtPAL-pdc-styABC*	glycerol	0.05 ± 0.00 (24–72 h)	2.13 ± 0.03 (80 h)	3.31 ± 0.01 (168 h)	0.019 ± 0.000 (0–168 h)	23.0 ± 0.1 (168 h)	81.8 ± 0.1 (168 h)	5B
4-hydroxybenzoate	GRC3Δ5-TYR3-*attTn7*:: *P*_*14f*_*-RtPAL-ech-vdh-fcs*	glycerol	0.05 ± 0.00 (24–74 h)	2.18 ± 0.03 (98 h)	6.19 ± 0.07 (98 h)	0.062 ± 0.001 (0–98 h)	34.8 ± 1.0 (98 h)	97.9 ± 0.2 (98 h)	5C

a “%” refers to carbon-molar percentage (% (Cmol/Cmol)).

b Product specificity refers to the target product titer's proportion of the sum of quantified aromatics, i.e., 4-coumarate, tyrosine, and *trans*-cinnamate as well as 4-vinylphenol, 4-hydroxyphenylacetate, and 4-hydroxybenzoate for the respective producers.

For microbial 4-hydroxyphenylacetate production, several biosynthetic pathways are available (Shen et al., 2019) that all involve 4-hydroxyphenylacetaldehyde as intermediate that is readily oxidized in *Pseudomonas* to the acid by native enzymes. Specifically, tyrosine can be converted to 4-hydroxyphenylacetaldehyde, either directly or via tyramine as intermediate. Alternatively, tyrosine's precursor 4-hydroxyphenylpyruvate is converted to 4-hydroxyphenylacetaldehyde (Shen et al., 2019). Only one pathway involves 4-coumarate and relies on the conversion of its decarboxylation product 4-vinylphenol, applying a styrene-catabolic pathway (Zhao et al., 2021). Previous studies on *P. putida* already indicated that the styrene degradation pathway accepts 4-vinylphenol (4-hydroxystyrene) as substrate, although it did not induce the associated *sty* operon in *P. putida* S12 (Verhoef et al., 2009). Moreover, styrene monooxygenase (StyAB) and styrene oxide isomerase (StyC) from *P. putida* were applied to establish tyrosol production from 4-coumarate in *E. coli* via 4-hydroxyphenylacetaldeyde (Lai et al., 2022). To enable *de novo* 4-hydroxyphenylacetate production via 4-coumarate, the *styABC* genes from *P. taiwanensis* VLB120 wild type's megaplasmid pSTY were chromosomally integrated with *RtPAL* and *pdc* into the Tn7 site of GRC3Δ5-TYR3 that lacks the pSTY megaplasmid and thus the original *styABC* operon. The resulting strain, GRC3Δ5-TYR3-*attTn7*::*P*_*14f*_-*RtPAL*-*pdc-styABC*, produced 3.31 ± 0.01 mM 4-hydroxyphenylacetate from 40 mM glycerol (Fig. 5B, Table 2) with a yield of 23.0 ± 0.1% (Cmol/Cmol). This yield is lower than that previously achieved (∼36% (Cmol/Cmol)) with *E. coli* (over)expressing phenylpyruvate decarboxylase (ARO10 from *S. cerevisiae*) and phenylacetaldehyde dehydrogenase (FeaB from *E. coli*), although this was achieved in a medium containing considerable concentrations of tryptone and yeast extract (Shen et al., 2019). During the cultivation a transient accumulation of 4-coumarate was observed (Fig. 5B) similar to that during 4-vinylphenol production (Fig. 5A). Strikingly, the titer and yield of 4-hydroxyphenylacetate were profoundly lower than those of 4-vinylphenol, although the latter is the direct precursor in the pathway applied here. Possibly, the formation of 4-hydroxyphenylacetaldeyhde as imminent precursor of 4-hydroxyphenylacetate exerts a toxic effect as (aromatic) aldehydes are well-known to be highly toxic (Zaldivar et al., 1999). Of note, 4-vinylphenol was not fully converted (0.23 ± 0.00 mM) and a higher *trans*-cinnamate accumulation (0.50 ± 0.00 mM) compared to the 4-vinylphenol production experiment was observed, which contributed to a lower product formation. Episomal expression of *RtPAL-pdc-styABC* could not enhance 4-hydroxyphenylacetate production, instead having the opposite effect (Fig. S8). From 20 mM glucose, only 0.39 ± 0.01 mM 4-hydroxyphenylacetate was produced with a significant accumulation of 4-vinylphenol (1.90 ± 0.02 mM) (Fig. S7C). This could indicate catabolite repression of *styABC* when glucose is used as carbon source. In *Pseudomonas*, several aromatics degradation pathways including that of styrene are known to be controlled by carbon catabolite repression, in which the Crc protein is a key component (Morales et al., 2004; Moreno et al., 2010; Rojo, 2010). However, the production module's expression was driven by a constitutive promoter and the catabolite activity motifs recognized by Crc (AANAANAA) (Moreno et al., 2015) identified in the 5′-untranslated regions of *styA* and *styC* were removed and replaced by synthetic ribosome-binding sites. To further enhance 4-hydroxyphenylacetate production, the alternative pathways mentioned above will be explored in the future.

Finally, 4-hydroxybenzoate production was established using *ech-vdh-fcs* from *P. putida* KT2440. In contrast to *P. putida*, the ferulic acid degradation pathway encoded by these genes is naturally absent in *P. taiwanensis* VLB120 (Lenzen et al., 2019). In addition to the β-oxidative 4-hydroxybenzoate biosynthesis, there are alternative pathways. Specifically, 4-hydroxybenzoate can be derived from 4-hydroxyphenylpyruvate via 4-hydroxymandelate (Chen et al., 2021) or from chorismate through chorismate pyruvate-lyases (Kitade et al., 2018). The latter is energetically more favorable than the β-oxidative route and involves less enzymes. However, chorismate is a hub chemical of many essential aromatics whose biosynthetic pathways compete for chorismate supply. Thus extensive engineering would be required to down-tune the respective pathways either on a transcriptional (Fernández-Cabezón et al., 2022) or enzymatic (Wynands et al., 2018) level. Alternatively competing pathways can be deleted, which, however, results in auxotrophies and the need for supplementation of costly additives (Yu et al., 2016). For these reasons, and to make optimal use of the 4-coumarate platform strain, the β-oxidative route was used here to enable 4-hydroxybenzoate production.

The Δ*pobA* mutation – already present in GRC3Δ5-TYR3 – prevents 4-hydroxybenzoate degradation and allows its accumulation upon product module implementation. GRC3Δ5-TYR3-*attTn7*::*P*_*14f*_-*RtPAL-ech-vdh-fcs* reached a maximum 4-hydroxybenzoate concentration of 6.19 ± 0.07 mM and a corresponding yield of 34.8 ± 1.0% (Cmol/Cmol) after 96 h when glycerol was completely consumed (Fig. 5C, Table 2). A maximum volumetric productivity of 0.153 ± 0.004 mM h^−1^ was achieved between 74 and 98 h. These numbers are a considerable improvement compared to the previous 4-hydroxybenzoate-producing, plasmid-bearing *P. taiwanensis* VLB120 CL3.3 that achieved a titer of 5.1 mM and yield of 29.6% (Cmol/Cmol) on glycerol under very similar conditions (Lenzen et al., 2019).

Overall, high-yield production of 4-vinylphenol, 4-hydroxyphenylacetate, and 4-hydroxybenzoate was enabled by insertion of the respective product pathway module into the 4-coumarate *chassis*, thereby demonstrating its broad applicability as a platform strain. However, yields vary significantly for the different products, indicating that the inserted pathway affects upstream metabolic fluxes and thus challenging the classical *chassis*-module dogma.

## 3 Conclusion

In this study, we established a *P. taiwanensis* platform strain that efficiently produces 4-coumarate from renewable resources. Ammonia-lyase activity displayed a major bottleneck during 4-coumarate production when substrate-specific TALs were applied, resulting in incomplete conversion of *de novo*-synthesized tyrosine. Therefore, the identification and screening of improved TALs (Brack et al., 2022) remains an important research field to debottleneck tyrosine-specific deamination in the future. This limitation was alleviated through the application of an unspecific *Rt*PAL accepting phenylalanine and tyrosine as substrates (PAL/TAL). To limit deamination of phenylalanine to *trans*-cinnamate as unwanted byproduct, a PheA^P144S^ mutation was implemented to reduce the flux through the phenylalanine loop. This strategy somewhat reduced growth and the overall production of phenylpropanoids, but it was highly effective in limiting byproduct formation and increasing product specificity to >95%. The engineered platform strain allowed high-yield production of not only 4-coumarate, but also 4-vinylphenol, 4-hydroxyphenylacetate, and 4-hydroxybenzoate from glycerol – to our knowledge – with the highest yields so far achieved for any *Pseudomonas* cell factory, proving the broad applicability of *P. taiwanensis* GRC3Δ5-TYR3 as platform strain for the synthesis of *para*-hydroxylated aromatics.

This study focused on achieving high yields in a minimal medium without the addition of amino acids or complex components. In the future, fed-batch fermentations are envisioned to increase product titers and rates. In this context, tolerance engineering of *Pseudomonas* for enhanced growth performance in the presence of aromatic stressors might be promising, for which studies on the underlying genetic and molecular mechanisms are of high value (Calero et al., 2018; Mohamed et al., 2020). Alternatively or additionally, *in situ* product removal strategies might be required to overcome product inhibition or toxicity. This can include the application of solvents in biphasic fermentations (Combes et al., 2021; Rodriguez et al., 2021; Verhoef et al., 2009) or in-stream solid-liquid or membrane-assisted liquid-liquid extractions (Combes et al., 2022).

## 4 Experimental procedures

### 4.1 Media and culture conditions

Routinely, *E. coli* and *P. taiwanensis* were grown in liquid lysogeny broth (LB) medium with 10 g L^−1^ tryptone, 5 g L^−1^ yeast extract, and 5 g L^−1^ sodium chloride, or on solid plates with LB that additionally contained 15 g L^−1^ agar (Carl Roth). Production experiments were performed using mineral salts medium (MSM) adapted from Hartmans et al. (1989) with a standard phosphate buffer capacity of 22.3 mM K_2_HPO_4_ and 13.6 mM NaH_2_PO_4_. In experiments for 4-coumarate, 4-hydroxybenzoate, and 4-hydroxyphenylacetate production, the buffer concentration was further increased two-fold. The initial 4-coumarate production experiment was performed with three-fold-buffered medium. d-(+)-Glucose monohydrate (Carl Roth) and glycerol (Chemsolute, Th. Geyer) served as carbon sources for production experiments. Pre-cultures contained the same carbon source as the main culture unless stated otherwise. *trans*-Ferulic acid (Carbosynth) and 4-coumaric acid (Sigma-Aldrich) were used as carbon sources to assess growth on these molecules. The pre-culture medium for the Growth Profiler experiment associated to Fig. S2 and Fig. S3 was supplemented with 0.1 mM phenylalanine to support similar growth. Incubation temperatures were 30 °C for *P. taiwanensis* and 37 °C for *E. coli*. Antibiotics were added to the media to ensure selective conditions as required but were omitted in main cultures for strains with chromosomal resistance markers. Kanamycin sulfate (Carl Roth) was used at a final concentration of 50 mg L^−1^. Gentamicin (Carl Roth) was applied at 10 mg L^−1^ in liquid and 25 mg L^−1^ in solid medium. *E. coli* DH5α λpir pTNS1 was grown on LB agar with 100 mg L^−1^ ampicillin sodium salt (Carl Roth). Sodium salicylate (Carl Roth) was used as an inducer for the *nagR*/*P*_*nagAa*_ expression system of pJNNopt- and pJNTmcs(t)-derived plasmids at a concentration of 0.1 mM. For the selection of *Pseudomonas*, cetrimide agar (Sigma-Aldrich) with 10 g L^−1^ glycerol or LB agar plates with 25 mg L^−1^ triclosan (Irgasan, Sigma-Aldrich) were used.

Liquid cultures were routinely grown in Erlenmeyer shake flasks with a filling volume of 10% (v/v) in a horizontal rotary shaker at 200 rpm with a throw of 50 mm. Small-scale cultivations (1.5 mL) were performed using System Duetz 24-well microtiter plates (CR1424a, EnzyScreen) sealed with sandwich covers (CR1224b, EnzyScreen) that were shaken at 300 rpm with a throw of 50 mm. 4-Vinylphenol production experiments were performed using 240-mL amber screw cap “Boston” bottles (Sigma-Aldrich, SKU #23235) with Mininert valves (Sigma-Aldrich, SKU #33304) filled with 20 mL culture. The initial OD_600_ was set to ∼0.05 in production and growth experiments.

Cultivations in the Growth Profiler 960 were performed using greyish-white 96-half-deep-well microtiter plates with flat transparent bottoms (CR1496dg, EnzyScreen) filled with 200 μL of cell suspensions at an initial OD_600_ of ∼0.05, sealed with sandwich covers (CR1296b, EnzyScreen), and shaken with 225 rpm.

### 4.2 Plasmid cloning and strain engineering

All strains used and generated in this study can be found in Table 3, and all plasmids in Table S1. Plasmids were constructed using the NEBuilder HiFi DNA Assembly Master Mix (New England Biolabs), applying the principle of Gibson cloning (Gibson et al., 2009). Further information on the cloning procedures of respective plasmids is provided in the Supplemental Information in Table S2. DNA-modifying enzymes were purchased from New England Biolabs. Primers for diagnostic PCRs, sequencing, and cloning were ordered from Eurofins Genomics as unmodified oligonucleotides. Used cloning primers are listed in the Supporting Information (Table S3). *StsTAL* was codon-optimized for *P. taiwanensis* VLB120 and ordered as gBlocks Gene Fragment (Integrated DNA Technologies). Codon-optimization was done using the OPTIMIZER online tool (Puigbò et al., 2007) as outlined in Wynands et al. (2018). *Fj*TAL, *tyrA*^fbr^, and *aroG*^fbr^ were previously codon-optimized for *P. taiwanensis* VLB120 using the same workflow (Lenzen et al., 2019; Wynands et al., 2018). The associated sequences can be found in Table S4. *RsTAL*, *HaTAL1*, and *SeSam8* were codon-optimized for *E. coli* (Jendresen et al., 2015). The RBS calculator v2.1 was used to predict or control translation initiation rates for (synthetic) ribosome binding sites (Salis et al., 2009).

**Table 3:** Bacterial strains used in this study.

Strain	Relevant characteristics	Reference
Escherichia coli		
DH5α	F^−^ Φ80 *lac*ZΔM15 Δ(*lac*ZYA-*arg*F)*U169 recA1 endA1 hsdR17*(r_k_^−^, m_k_^+^) *phoA supE44 thi-1 gyrA96 relA1* λ^−^	Thermo Fischer Scientific
DH5α λ*pir*	λ*pir* lysogen of DH5α; host for *oriV(R6K)* plasmids	Platt et al. (2000)
PIR2	*F*^*−*^ Δ*lac169 rpoS(Am) robA1 creC510 hsdR514 endA recA1 uidA(*Δ*MluI)*:*pir*; host for *oriV(R6K)* plasmids	Thermo Fischer Scientific
EC100D *pir*+	F^*−*^*mcrA* Δ*(mrr-hsdRMS-mcrBC)* Φ80d*lacZ*ΔM15 Δ*lacX74 recA1 endA1 araD139* Δ*(ara, leu)7697 galU galK λ*^*–*^*rpsL nupG pir*^+^*(DHFR)*; host for *oriV(R6K)* plasmids	Lucigen
HB101 pRK2013	HB101 with pRK2013	Ditta et al. (1980)
DH5α λ*pir* pTNS1	DH5α λ*pir*with pTNS1	Choi et al. (2005)
DH5α pSW-2	DH5α with pSW-2	Martinez-Garcia and de Lorenzo (2011)
***Pseudomonas putida***		
KT2440	wild-type strain derived from *Pseudomonas putida* mt-2 through loss of the TOL plasmid pWW0	Bagdasarian et al. (1981) MiKat#30
S12 C3	4-coumarate producer obtained by *N*-methyl-*N*′-nitro-*N*-nitrosoguanidine mutagenesis, antimetabolite selection, high-throughput screening, targeted disruption of *fcs* (*fcs*::*tetA*), and episomal expression of the *Rt*PAL using plasmid pTac*pal*	Nijkamp et al. (2007)
***Pseudomonas taiwanensis***		
VLB120	wild-type strain	Panke et al. (1998) MiKat#1
GRC3	genome-reduced *chassis* strain of VLB120 with ΔpSTY, Δprophage1/2:*ttgVWGHI*, Δprophage3, Δprophage4, Δflag1, Δflag2, Δlap1, Δlap2, Δlap3	Wynands et al. (2019) MiKat#5
GRC3*-attTn7*::*P*_*14f*_*-ech-vdh-fcs*	GRC3 with *attTn7*::*Kan_FRT_P*_*14f*_-*ech-vdh-fcs*	This study MiKat#2197
GRC3Δ5-TYR1	GRC3, Δ*pobA*, Δ*hpd*, Δ*quiC*, Δ*quiC1*, Δ*quiC2*, *trpE*^P290S^, *aroF-1*^P148L^, Δ*pykA*	Wynands et al. (2019) MiKat#660
GRC3Δ5-TYR2	GRC3Δ5-TYR1 with *pheA*^T310I^	Wynands et al. (2019) MiKat#58
GRC3Δ5-TYR3	GRC3Δ5-TYR2 with *pheA*^P144S^	This study MiKat#60
GRC3Δ5-TYR4	GRC3Δ5-TYR1 with *pheA*^P144S^	This study MiKat#729
GRC3Δ5-TYR1.1	GRC3Δ5-TYR1 with PVLB_23545/40:*P*_*14e*_-*tyrA*^fbr^	This study MiKat#732
GRC3Δ5-TYR2.1	GRC3Δ5-TYR2 with PVLB_23545/40:*P*_*14e*_-*tyrA*^fbr^	This study MiKat#734
GRC3Δ5-TYR3.1	GRC3Δ5-TYR3 with PVLB_23545/40:*P*_*14e*_-*tyrA*^fbr^	This study MiKat#736
GRC3Δ5-TYR4.1	GRC3Δ5-TYR4 with PVLB_23545/40:*P*_*14e*_-*tyrA*^fbr^	This study MiKat#768
GRC3Δ5-TYR1.2	GRC3Δ5-TYR1 with PVLB_23545/40:*P*_*14e*_-*tyrA*^fbr^*-aroG*^fbr^	This study MiKat#1112
GRC3Δ5-TYR2.2	GRC3Δ5-TYR2 with PVLB_23545/40:*P*_*14e*_-*tyrA*^fbr^*-aroG*^fbr^	This study MiKat#1114
GRC3Δ5-TYR3.2	GRC3Δ5-TYR3 with PVLB_23545/40:*P*_*14e*_-*tyrA*^fbr^*-aroG*^fbr^	This study MiKat#1116
GRC3Δ5-TYR4.2	GRC3Δ5-TYR4 with PVLB_23545/40:*P*_*14e*_-*tyrA*^fbr^*-aroG*^fbr^	This study MiKat#1118
GRC3Δ5-TYR2-*attTn7*::*P*_*14f*_*-RsTAL*	GRC3Δ5-TYR2 with *attTn7*::*Kan_FRT_P*_*14f*_-*RsTAL*	This study MiKat#1086
GRC3Δ5-TYR2-*attTn7*::*P*_*14f*_*-HaTAL1*	GRC3Δ5-TYR2 with *attTn7*::*Kan_FRT_P*_*14f*_-*HaTAL1*	This study MiKat#1084
GRC3Δ5-TYR2-*attTn7*::*P*_*14f*_*-SeSam8*	GRC3Δ5-TYR2 with *attTn7*::*Kan_FRT_P*_*14f*_-*SeSam8*	This study MiKat#1085
GRC3Δ5-TYR2-*attTn7*::*P*_*14f*_*-FjTAL*	GRC3Δ5-TYR2 with *attTn7*::*Kan_FRT_P*_*14f*_-*FjTAL*	This study MiKat#1087
GRC3Δ5-TYR2 pJNNopt-*FjTAL*	GRC3Δ5-TYR2 with pJNNopt-*FjTAL*	This study MiKat#1091
GRC3Δ5-TYR2-*attTn7*::*P*_*14f*_*-StsTAL*	GRC3Δ5-TYR2 with *attTn7*::*Kan_FRT_P*_*14f*_*-StsTAL*	This study MiKat#1088
GRC3Δ5-TYR2 pJNNopt-*StsTAL*	GRC3Δ5-TYR2 with pJNNopt-*StsTAL*	This study MiKat#1092
GRC3Δ5-TYR1-*attTn7*::*P*_*14f*_*-RtPAL*	GRC3Δ5-TYR1 with *attTn7*::*Kan_FRT_P*_*14f*_-*RtPAL*	This study MiKat#969
GRC3Δ5-TYR1.1-*attTn7*::*P*_*14f*_*-RtPAL*	GRC3Δ5-TYR1.1 with *attTn7*::*Kan_FRT_P*_*14f*_-*RtPAL*	This study MiKat#1467
GRC3Δ5TYR1.2-*attTn7*::*P*_*14f*_*-RtPAL*	GRC3Δ5-TYR1.2 with *attTn7*::*Kan_FRT_P*_*14f*_-*RtPAL*	This study MiKat#1382
GRC3Δ5-TYR2-*attTn7*::*P*_*14f*_*-RtPAL*	GRC3Δ5-TYR2 with *attTn7*::*Kan_FRT_P*_*14f*_-*RtPAL*	This study MiKat#958
GRC3Δ5-TYR2 pJNNopt-*RtPAL*	GRC3Δ5-TYR2 with pJNNopt-*RtPAL*	This study MiKat#1089
GRC3Δ5-TYR2.1-*attTn7*::*P*_*14f*_*-RtPAL*	GRC3Δ5-TYR2.1 with *attTn7*::*Kan_FRT_P*_*14f*_-*RtPAL*	This study MiKat#1468
GRC3Δ5-TYR2.2-*attTn7*::*P*_*14f*_*-RtPAL*	GRC3Δ5-TYR2.2 with *attTn7*::*Kan_FRT_P*_*14f*_-*RtPAL*	This study MiKat#1383
GRC3Δ5-TYR3-*attTn7*::*P*_*14f*_*-RtPAL*	GRC3Δ5-TYR3 with *attTn7*::*Kan_FRT_P*_*14f*_-*RtPAL*	This study MiKat#959
GRC3Δ5-TYR3 pJNNopt-*RtPAL*	GRC3Δ5-TYR3 with pJNNopt-*RtPAL*	This study MiKat#1090
GRC3Δ5-TYR4-*attTn7*::*P*_*14f*_*-RtPAL*	GRC3Δ5-TYR4 with *attTn7*::*Kan_FRT_P*_*14f*_-*RtPAL*	This study MiKat#970
GRC3Δ5-TYR4.1-*attTn7*::*P*_*14f*_*-RtPAL*	GRC3Δ5-TYR4.1 with *attTn7*::*Kan_FRT_P*_*14f*_-*RtPAL*	This study MiKat#1168
GRC3Δ5-TYR4.1 pJNNopt-*RtPAL*	GRC3Δ5-TYR4.1 with pJNNopt-*RtPAL*	This study MiKat#1381
GRC3Δ5-TYR4.2-*attTn7*::*P*_*14f*_*-RtPAL*	GRC3Δ5-TYR4.2 with *attTn7*::*Kan_FRT_P*_*14f*_-*RtPAL*	This study MiKat#1385
GRC3Δ5-TYR4.2 pJNNopt-*RtPAL*	GRC3Δ5-TYR4.2 with pJNNopt-*RtPAL*	This study MiKat#1380
GRC3Δ5-TYR3-*attTn7*::*P*_*14f*_*-RtPAL-pdc*	GRC3Δ5-TYR3 with *attTn7*::*Kan_FRT_P*_*14f*_-*RtPAL-pdc*	This study MiKat#961
GRC3Δ5-TYR3-*attTn7*::*P*_*14f*_*-RtPAL-pdc-styABC*	GRC3Δ5-TYR3 with *attTn7*::*Kan_FRT_P*_*14f*_-*RtPAL-pdc-styABC*	This study MiKat#1663
GRC3Δ5-TYR3 pJNNopt-*RtPAL-pdc-styABC*	GRC3Δ5-TYR3 with pJNNopt-*RtPAL-pdc-styABC*	This study MiKat#1388
GRC3Δ5-TYR3 pJNT*palpdc-styABC*	GRC3Δ5-TYR3 with pJNT*palpdc-styABC*	This study MiKat#1533
GRC3Δ5-TYR3-*attTn7*::*P*_*14f*_*-RtPAL-ech-vdh-fcs*	GRC3Δ5-TYR3 with *attTn7*::*Kan_FRT_P*_*14f*_-*RtPAL-ech-vdh-fcs*	This study MiKat#960

PCRs for the amplification of DNA used for cloning were performed using the Q5 High-Fidelity 2X Master Mix (New England Biolabs) according to the manufacturer's instruction manual. Diagnostic colony PCRs were done with the One*Taq* Quick-Load 2X Master Mix with Standard Buffer (New England Biolabs). Template colonies were pre-lyzed with alkaline PEG 200, according to Chomczynski and Rymaszewski (2006).

Electroporation was performed using a GenePulser Xcell (BioRad) with the following settings: 2 mm cuvette gap, 2.5 kV, 200 Ω, 25 μF. Electrocompetent *Pseudomonas* cells were prepared according to a protocol adapted from Choi et al. (2005).

For the conjugational transfer of mobilizable plasmids, patch matings were performed as described in Wynands et al. (2018). Briefly, all required strains were streaked above each other onto an LB plate and incubated for several hours at 30 °C, then an inoculation loop of the mating lawn was resuspended in 1 mL 0.9% (w/v) sodium chloride, of which proportions were spread onto selective agar plates.

The *pheA* mutation (P144S; CCG→AGC) was introduced using the pEMG/pSW-2 system (Martinez-Garcia and de Lorenzo, 2011) applying the workflow described in Wynands et al. (2018). The chromosomal integration of *tyrA*^fbr^ and *aroG*^fbr^ was achieved by the same technique using pEMG-PVLB_23545/40-*P*_*14e*_*-tyrA*^fbr^ or pEMG-PVLB_23545/40-*P*_*14e*_*-tyrA*^fbr^*-aroG*^fbr^.

For the delivery of deletion/integration plasmids, the *Pseudomonas* recipient, the helping strain *E. coli* HB101 pRK2013, and the *E. coli* donor were patched for conjugation. For the delivery of mini-Tn7 transposons into the *attTn7* site, the *Pseudomonas* recipient, the helping strain *E. coli* HB101 pRK2013, the *E. coli* donor, and *E. coli* DH5α λ*pir* pTNS1 that provides the required transposase proteins *in trans* were patched. Genomic modifications were mapped by colony PCR and sequenced when necessary, i.e., in case of introduced point mutations.

### 4.3 Analytical methods

Optical densities of cell suspensions were measured at a wavelength of 600 nm (OD_600_) using an Ultrospec 10 photometer (Biochrom). Online growth curves were obtained using the Growth Profiler 960 and the corresponding GP960Viewer software (EnzyScreen). The Growth Profiler 960 captures bottom-up images of transparent-bottom microtiter plates and determines the green value that correlates with optical densities in a non-linear manner.

For the detection and quantification of substrates, metabolites, and products, cell culture supernatants were sampled by centrifugation, filtered, and stored at −20 °C until they were analyzed by HPLC in a 1260 Infinity II system equipped with a 1260 DAD WR and 1260 RI detector (Agilent Technologies). Aromatic compounds were analyzed using a reversed-phase HPLC column, InfinityLab Poroshell 120 EC-C18 (3.0 × 150 mm, 2.7 μm, Agilent Technologies, P.N. 693975-302T) with guard column (Agilent Technologies; P.N.: 823750-911) or ISAspher 100-5 C18 BDS (4.0 × 250 mm, ISERA; P.N.: A111-C25S00) with guard column (ISERA; P.N.: A112-C25G30), that were eluted in a gradient with 0.1% (v/v) trifluoroacetic acid (TFA, Sigma-Aldrich) and acetonitrile (Th. Geyer) at a flow rate of 0.8 mL min^−1^ and a temperature of 40 °C. The column-specific elution profiles are shown in the Supporting information (Table S5 and Table S6). *trans*-Cinnamate and 4-coumarate were detected and analyzed at wavelengths of 240 and 260, respectively, tyrosine, 4-vinylphenol, 4-hydroxyphenylacetate, and 4-hydroxybenzoate at 280 nm. Solutions of l-tyrosine (≥99%, Sigma-Aldrich), 4-coumaric acid (≥98%, Sigma-Aldrich), *trans*-cinnamic acid (≥99%, Sigma-Aldrich), 4-hydroxybenzoic acid (99%, Sigma-Aldrich), 4-vinylphenol (10% w/v in propylene glycol, Sigma-Aldrich), and 4-hydroxyphenylacetic acid (98%, Sigma-Aldrich) served as authentic analytical standards.

d-Glucose, d-gluconate, and glycerol concentrations were determined using a Luna Omega 3 μm SUGAR 100 Å (150 × 4.6 mm, Phenomenex; P.N.: 00F-4775-E0) column equipped with a SecurityGuard (Phenomenex; P.N.: KJ0-4282) and Guard cartridge (SUGAR, 4 × 3.0 mm ID, Phenomenex; P.N.: AJ0-4495). This column was eluted isocratically with 20 mM KH_2_PO_4_ in 3% (v/v) methanol (Th. Geyer) with an adjusted pH of 2.5 at a flow rate of 0.5 mL min^−1^ and a temperature of 25 °C for 15 min. Alternatively, glycerol concentrations were measured using a Metab-AAC (300 × 7.8 mm, ISERA; P.N.: A1BF-A1AA0N) column equipped with a Guard Cartridge Holder (ISERA, P.N.: AA13-000005) and Guard Column (10 × 7.8 mm, ISERA; A1BF-A1AG0N) that was eluted for 20 min with 5 mM H_2_SO_4_ at a flow of 0.6 mL min^−1^ and a temperature of 50 °C. Glucose, gluconate, and glycerol were analyzed using the RID. d-(+)-Glucose monohydrate (≥99.5%, Carl Roth), d-gluconic acid sodium salt (Sigma-Aldrich), and glycerol (Chemsolute, ≥99%, Th. Geyer) served as analytical standards.

Product yields were calculated considering the initial and maximum product concentrations and the consumed carbon source. The growth rates shown in Table 1, Table 2 were determined applying linear fits (with R^2^ ≥ 0.98) to the natural logarithmic values of the available OD_600_ data points (≥3) of the indicated time ranges.

All experiments were performed in biological replicates as indicated. Rare analytical outliers were excluded from data sets. Errors indicate standard deviations. Statistical significance was assessed by *t*-test (two-tailed distribution, heteroscedastic, *p* < 0.05).

## Funding

We acknowledge funding from the European Union's Horizon 2020 research and innovation program (grant agreement number 953073) for the UPLIFT project and from the German 10.13039/501100002347Federal Ministry of Education and Research (BMBF) for the NO-STRESS project (FKZ 031B0852A).

## CRediT author statement

**B. Wynands:** Conceptualization, Investigation, Validation, Supervision, Visualization, Writing – Original Draft; **F. Kofler:** Investigation, Validation; **A. Sieberichs:** Investigation; **N. da Silva:** Investigation, Validation; **N. Wierckx:** Conceptualization, Funding acquisition, Supervision, Writing – Review & Editing.

## Declaration of competing interest

The authors declare no competing interest.

## Data Availability

Data will be made available on request.
