# Supplementary material for: Engineering a Pseudomonas taiwanensis 4-coumarate platform for production of para-hydroxy aromatics with high yield and specificity
Source: Metab Eng. 2023 Jul;78:115–27. doi: 10.1016/j.ymben.2023.05.004 (PMC10360455; doi:10.1016/j.ymben.2023.05.004)
Supplement: Multimedia component 1 [file mmc1.pdf]

Supplementary material to the article "Engineering a *Pseudomonas taiwanensis* 4-coumarate platform for production of *para*-hydroxy aromatics with high yield and specificity"

Benedikt Wynands[a], Franziska Kofler[a], Anka Sieberichs[a], Nadine da Silva[a], and Nick Wierckx[a*]

[a]Institute of Bio- and Geosciences, IBG-1: Biotechnology, Forschungszentrum Jülich GmbH, 52425 Jülich, Germany

* Corresponding author:

Nick Wierckx, Institute of Bio- and Geosciences, IBG-1: Biotechnology, Forschungszentrum Jülich GmbH, 52425 Jülich, Germany, phone: +49 2461 61-85247, e-mail: n.wierckx@fz-juelich.de

Table of contents:

Fig. S1	page 2
Fig. S2	page 3
Fig. S3	page 4
Fig. S4	page 5
Fig. S5	page 5
Fig. S6	page 6
Fig. S7	page 7
Fig. S8	page 8
Table S1	page 9
Table S2	page 10
Table S3	page 11
Table S4	page 12
Table S5	page 13
Table S6	page 13
References	page 13

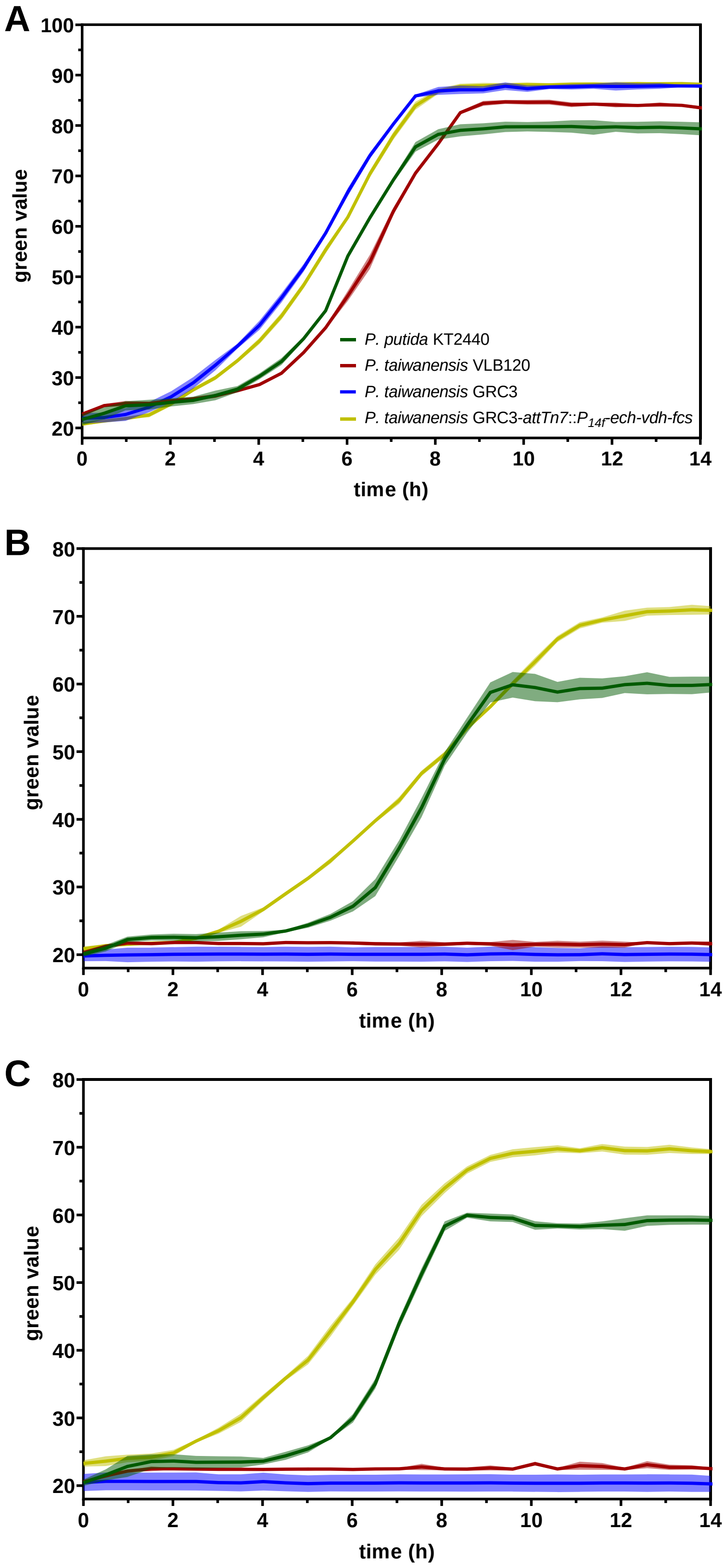

Fig. S1. Growth of *Pseudomonas putida* KT2440, *Pseudomonas taiwanensis* VLB120, *P. taiwanensis* GRC3, and *P. taiwanensis* GRC3-*attTn7*::*P*$_{14f}$-*ech-vdh-fcs* in MSM (one-fold-buffered) with 20 mM glucose (A), or MSM (two-fold-buffered) with 10 mM *trans*-ferulic acid (B), or 10 mM 4-coumaric acid (C) as sole carbon and energy source. Cultures were grown in the Growth Profiler 960. Shaded areas indicate the standard deviation of replicates (n = 4). Pre-cultures were grown in MSM with 20 mM glucose (one-fold-buffered).

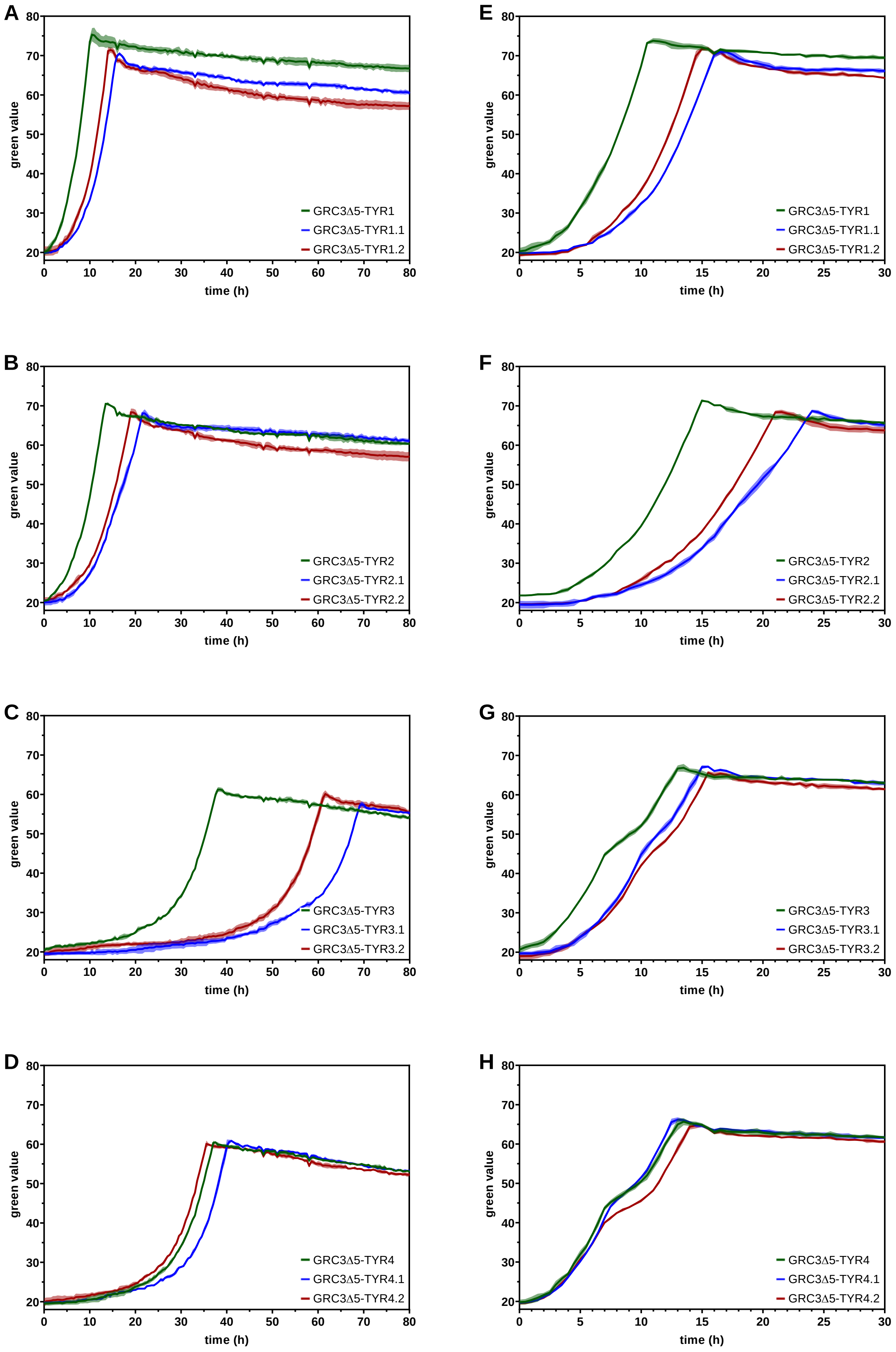

Fig. S2. Growth of tyrosine-producing *Pseudomonas taiwanensis* GRC3Δ5-derived strains in MSM with 20 mM glucose without (A, B, C, and D) and with supplementation of 0.1 mM phenylalanine (E, F, G, and H). Cultures were grown in the Growth Profiler 960. Shaded areas indicate the standard deviation of replicates (n = 4). Pre-cultures were grown in MSM with 20 mM glucose and 0.1 mM phenylalanine.

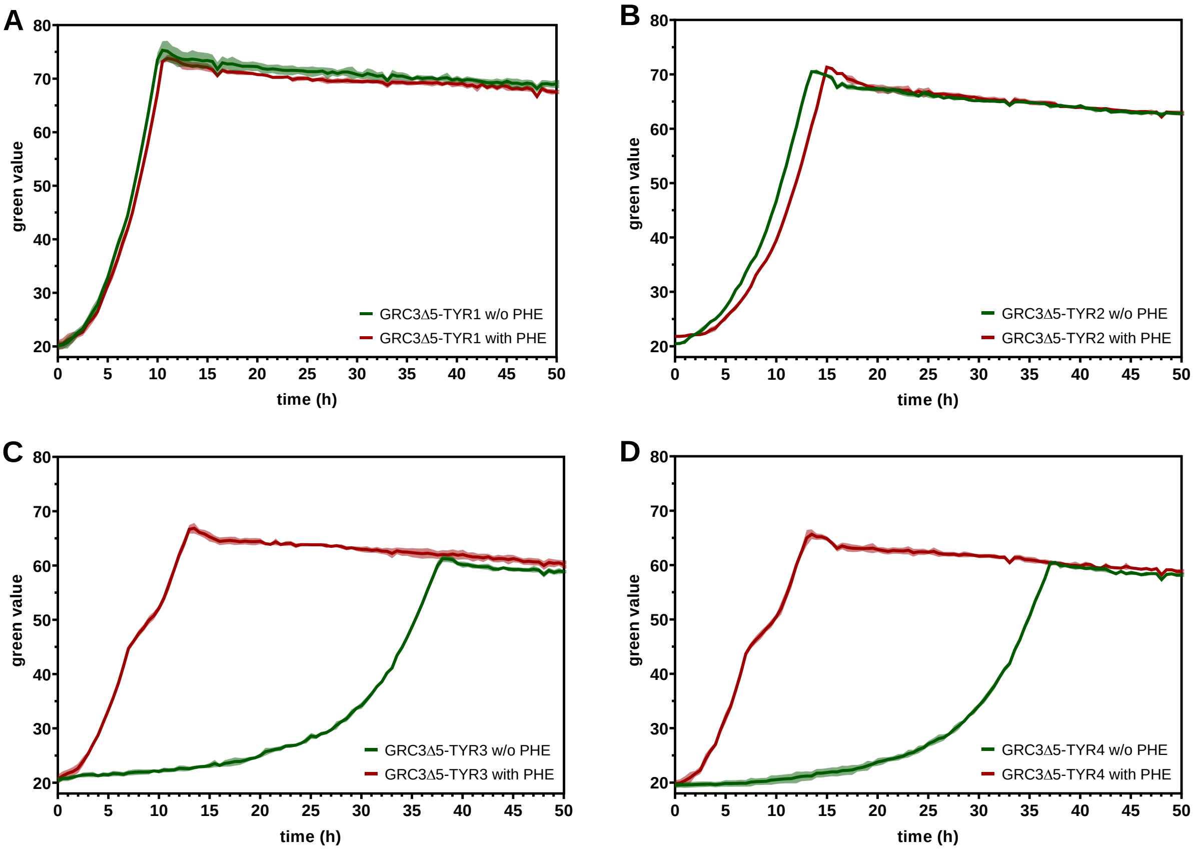

Fig. S3. Growth of tyrosine-producing *Pseudomonas taiwanensis* GRC3Δ5-TYR1 (A), TYR2 (B), TYR3 (C), and TYR4 (D) in MSM with 20 mM glucose without and with supplementation of 0.1 mM phenylalanine. The strains' growth profiles are those presented in Fig. S2. Cultures were grown in the Growth Profiler 960. Shaded areas indicate the standard deviation of replicates (n = 4). Pre-cultures were grown in MSM with 20 mM glucose and 0.1 mM phenylalanine. Abbreviations: PHE, phenylalanine; w/o, without.

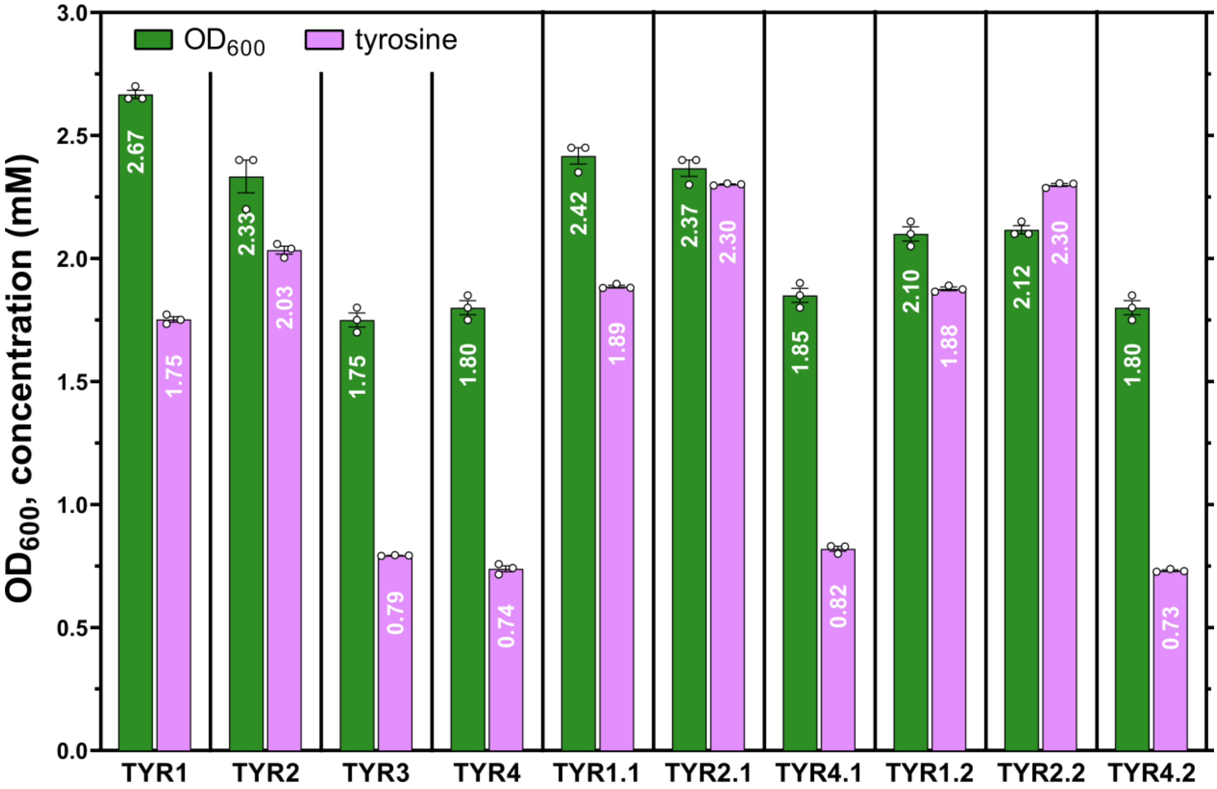

Fig. S4. OD_{600} and tyrosine formation of *Pseudomonas taiwanensis* GRC3Δ5-derived tyrosine producers. Cultures were grown in 24-well microtiter plates for 96 h using MSM with 20 mM glucose. Error bars indicate the standard deviation of triplicates (n = 3). Strains GRC3Δ5-TYR3.1 and GRC3Δ5-TYR4.1 are not shown because they did not fully consume the carbon source and reach stationary phase within 96 h under the tested conditions.

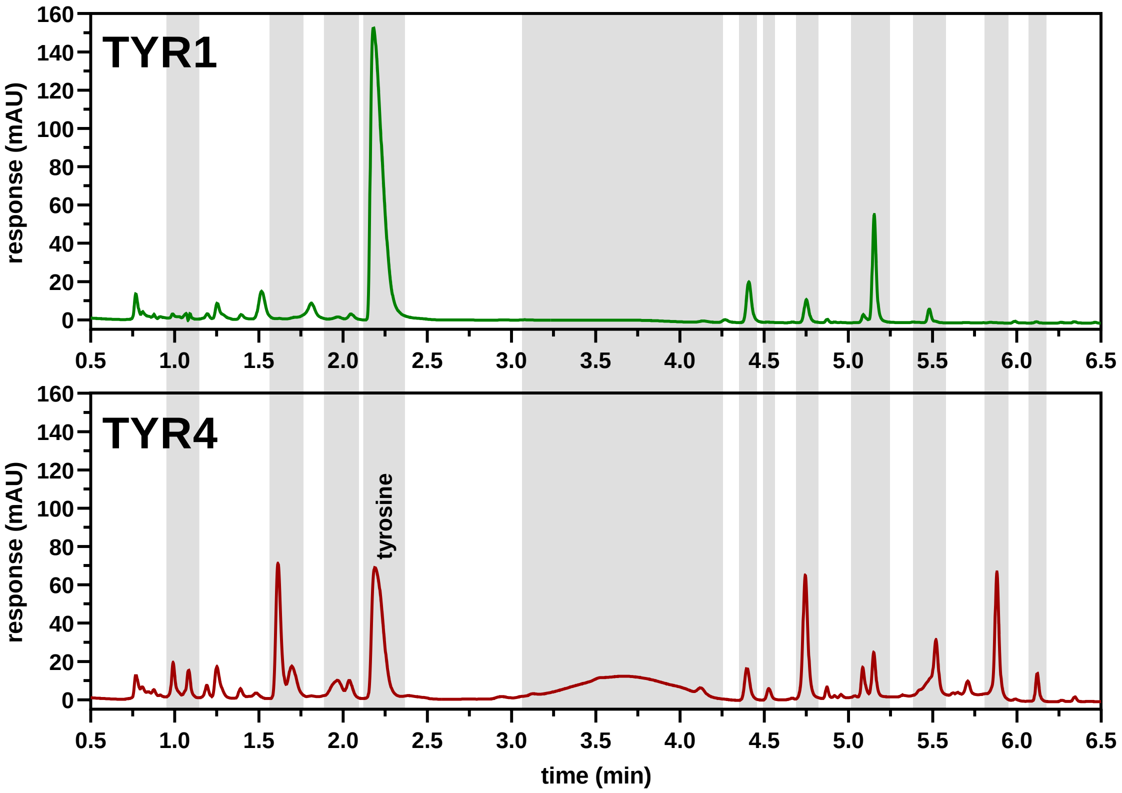

Fig. S5. Representative HPLC chromatograms with InfinityLab Poroshell 120 EC-C18 at 280 nm of tyrosine-producing *Pseudomonas taiwanensis* GRC3Δ5-TYR1 (*pheA*^{WT}) and TYR4 (*pheA*^{P144S}) after 96 h grown in MSM with 20 mM glucose.

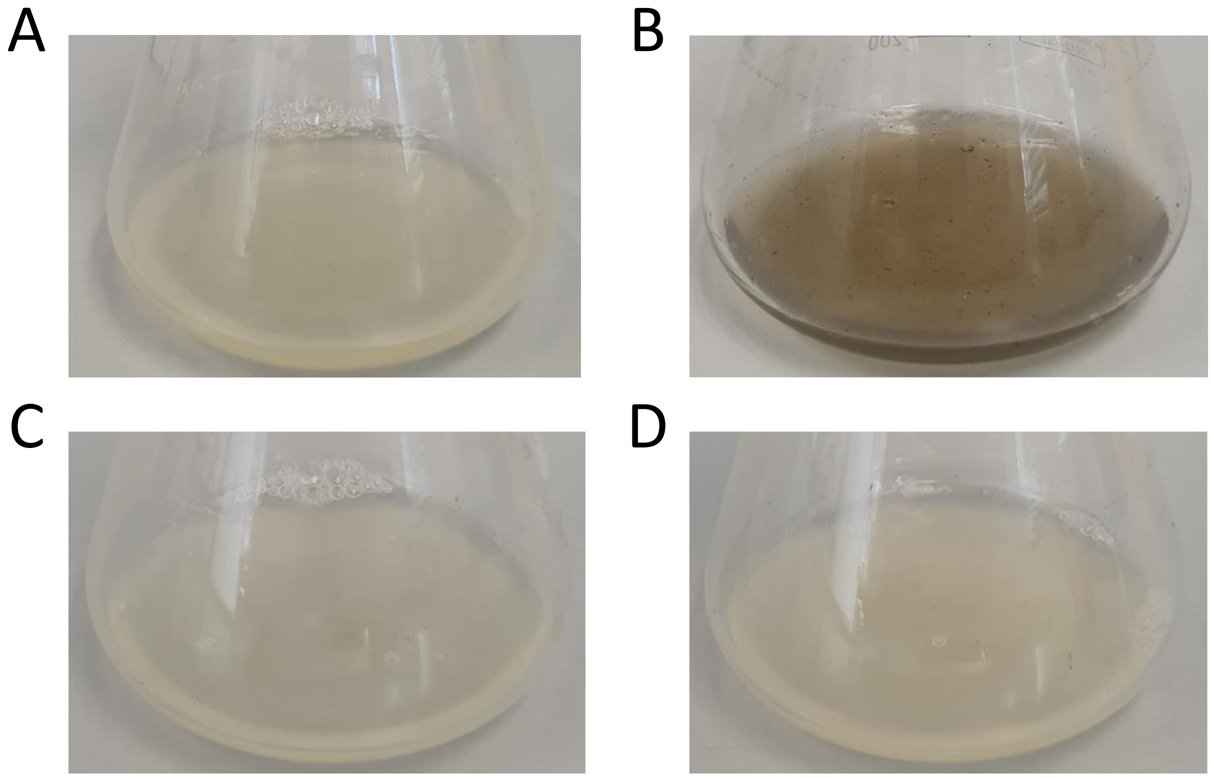

Fig. S6. Representative pictures of shake flasks with GRC3Δ5-TYR2 pJNNopt-*RtPAL* after 96 h on 20 mM glucose (A) and after 120 h on 40 mM glycerol (C) or GRC3Δ5-TYR3 pJNNopt-*RtPAL* after 96 h on 20 mM glucose (B) and after 120 h on 40 mM glycerol (D).

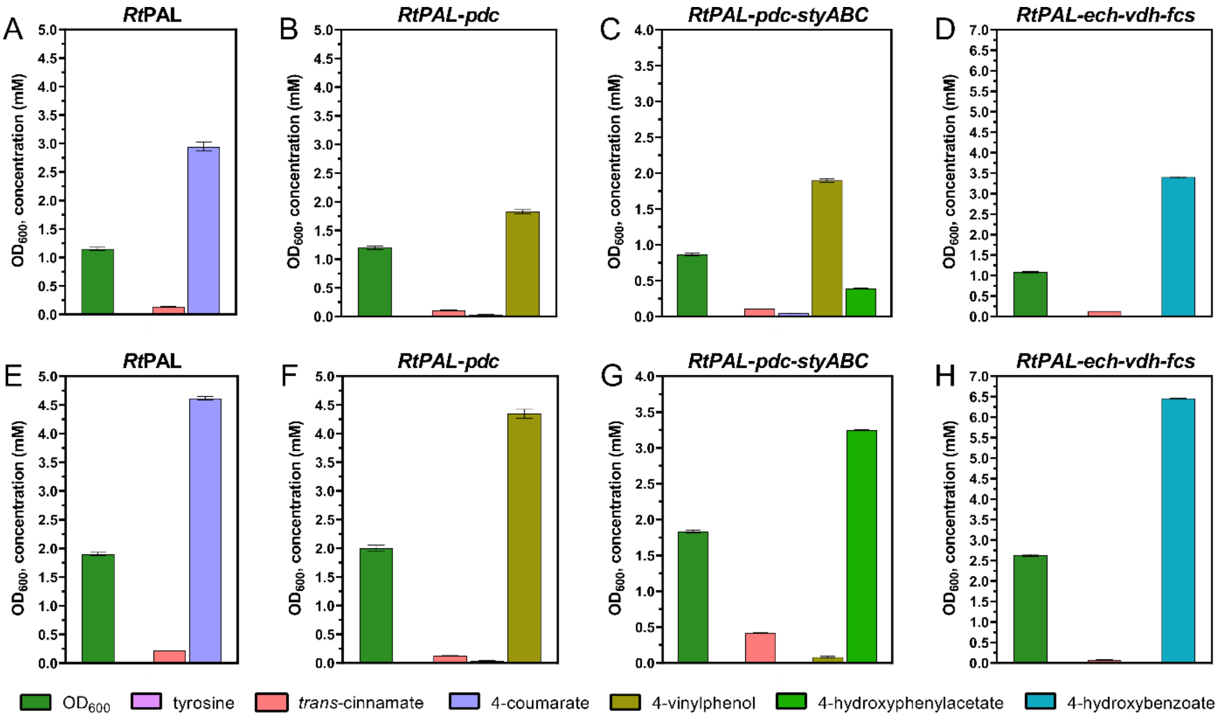

Fig. S7. Production of 4-coumarate with GRC3Δ5-TYR3-*attTn7*::*P14f-RtPAL* (A, E), 4-vinylphenol with GRC3Δ5-TYR3-*attTn7*::*P14f-RtPAL-pdc* (B, F), 4-hydroxyphenylacetate with GRC3Δ5-TYR3-*attTn7*::*P14f-RtPAL-pdc-styABC* (C, G), and 4-hyxdroxybenzoate with GRC3Δ5-TYR3-*attTn7*::*P14f-RtPAL-ech-vdh-fcs* (D, H) from either 20 mM glucose (A-D) or 40 mM glycerol (E-H) after 120 h. The cultivations on glycerol were performed as a control to confirm the strains' production performances associated to Fig. 5, for which similar titers were achieved with differences of <10%. MSM was one-fold-buffered for the production of 4-vinylphenol and two-fold-buffered for the other cultivations. 4-Coumarate and 4-hydroxybenzoate production was assessed in System Duetz 24-well microtiter plates, 4-vinylphenol in closed 240-mL amber screw cap bottles, and 4-hydroxyphenylacetate in 500-mL shake flasks. Error bars indicate the standard deviation of replicates (n ≥ 2).

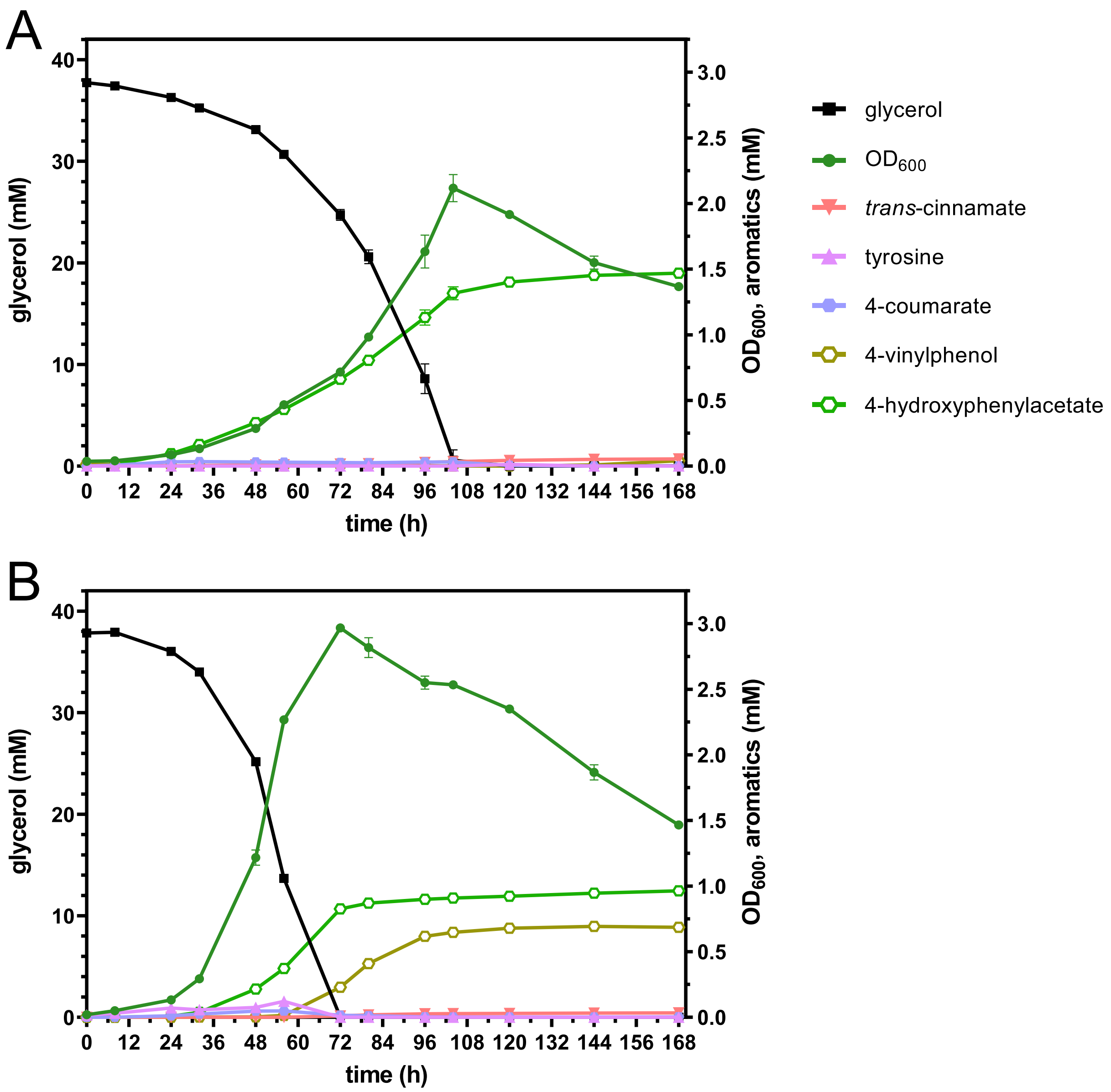

Fig. S8. Shake flask cultivations of GRC3Δ5-TYR3 with pJNNopt-*RtPAL-pdc-styABC* (A) or pJNT*palpdc-styABC* (B) cultivated in MSM with 40 mM glycerol (two-fold-buffered). Error bars indicate the standard deviation of replicates (n = 3).

Table S1 Plasmids used in this study with relevant characteristics and references.

Plasmids	Relevant characteristics	Reference
pRK2013	Km^R, *oriV(ColE1)*, mob^+, tra^+, mobilization helper plasmid for the conjugative transfer of mobilizable plasmids	Figurski and Helinski (1979)
pEMG	Km^R, *oriV(R6K)*, *oriT*, *traJ*, *lacZα*-MCS flanked by two I-SceI restriction sites	Martínez-García and de Lorenzo (2011)
pEMG-*pheA*^{P144S}	pEMG-derived replacement plasmid for generation of *pheA*^{P144S}, harbors the fused flanking regions of codon 144 (CCG) linked by the replacement codon (AGC)	This study
pEMG-PVLB_23545/40-P_{14e}-*tyrA*^{fbr}	pEMG-derived plasmid for insertion of P_{14e}-*tyrA*^{fbr} into the intergenic locus PVLB_23545/40	This study
pEMG-PVLB_23545/40-P_{14e}-*tyrA*^{fbr}-*aroG*^{fbr}	pEMG-derived plasmid for insertion of P_{14e}-*tyrA*^{fbr}-*aroG*^{fbr} into the intergenic locus PVLB_23545/40	This study
pSW-2	Gm^R, *oriV(RK2)*, *oriT*, *xylS*/P_m→*I-sceI*	Martínez-García and de Lorenzo (2011)
pBG14f_*FRT_Kan*	Km^R flanked by FRT sites, *oriV(R6K)*, *oriT*, P_{14f}→*msfgfp*, mini-Tn7 transposon plasmid	Ackermann et al. (2021)
pTN1_*nagR*/P_{nagAa}_Opt_GFP (here denoted pJNNopt-*gfp*)	Gm^R, Ap^R, *oriV(ColE1)*, *oriV(pRO1600)*, *nagR*/P_{NagAa}→*msfgfp*	Neves et al. (2019)
pBG14f_*FRT_Kan_RsTAL*	pBG14f_*FRT_Kan*-derived plasmid for Tn7 transposition, P_{14f}→*RsTAL*	This study
pBG14f_*FRT_Kan_HaTAL1*	pBG14f_*FRT_Kan*-derived plasmid for Tn7 transposition, P_{14f}→*HaTAL1*	This study
pBG14f_*FRT_Kan_SeSam8*	pBG14f_*FRT_Kan*-derived plasmid for Tn7 transposition, P_{14f}→*SeSam8*	This study
pBG14f_*FRT_Kan_FjTAL*	pBG14f_*FRT_Kan*-derived plasmid for Tn7 transposition, P_{14f}→*FjTAL*	This study
pJNNopt-*FjTAL*	pJNNopt-derived plasmid with *nagR*/P_{NagAa}→*FjTAL*	This study
pBG14f_*FRT_Kan_StsTAL*	pBG14f_*FRT_Kan*-derived plasmid for Tn7 transposition, P_{14f}→*StsTAL*	This study
pJNNopt-*StsTAL*	pJNNopt-derived plasmid with *nagR*/P_{NagAa}→*StsTAL*	This study
pBG14f_*FRT_Kan_RtPAL*	pBG14f_*FRT_Kan*-derived plasmid for Tn7 transposition, P_{14f}→*RtPAL*	This study
pJNNopt-*RtPAL*	pJNNopt-derived plasmid with *nagR*/P_{NagAa}→*RtPAL*	This study
pBG14f_*FRT_Kan_RtPAL-pdc*	pBG14f_*FRT_Kan*-derived plasmid for Tn7 transposition, P_{14f}→*RtPAL-pdc*	This study
pJNT*palpdc*	Gm^R, Ap^R, *oriV(ColE1)*, *oriV(pRO1600)*, *nagR*/P_{NagAa}→*RtPAL-pdc*; pJNTmcs(t)-derived plasmid	Verhoef et al. (2009)
pBG14f_*FRT_Kan_RtPAL-pdc-styABC*	pBG14f_*FRT_Kan*-derived plasmid for Tn7 transposition, P_{14f}→*RtPAL-pdc-styABC*	This study
pJNT*palpdc-styABC*	Gm^R, Ap^R, *oriV(ColE1)*, *oriV(pRO1600)*, *nagR*/P_{NagAa}→*RtPAL-pdc-styABC*; pJNTmcs(t)-derived plasmid	This study
pJNNopt-*RtPAL-pdc-styABC*	pJNNopt-derived plasmid with *nagR*/P_{NagAa}→*RtPAL-pdc-styABC*	This study
pBG14f_*FRT_Kan_RtPAL-ech-vdh-fcs*	pBG14f_*FRT_Kan*-derived plasmid for Tn7 transposition, P_{14f}→*RtPAL-ech-vdh-fcs*	This study
pBG14f_*FRT_Kan_ech-vdh-fcs*	pBG14f_*FRT_Kan*-derived plasmid for Tn7 transposition, P_{14f}→*ech-vdh-fcs*	unpublished
pTNS1	Ap^R, *oriV(R6K)*, mob^+, TnsABC+D expression plasmid for Tn7 site-specific transposition	Choi et al. (2005)

Abbreviations: Ap^R, ampicillin resistance; Gm^R, gentamicin resistance; Km^R, kanamycin resistance.

Table S2 Details on plasmid cloning procedures.

Plasmid name	Cloning procedure
pEMG-*pheA*^{P144S}	pEMG was digested using SacI-HF and SalI-HF. TS1-*pheA*^{P144S} and TS2-*pheA*^{P144S} were PCR-amplified with primers BW539/BW540 and BW541/BW542, respectively, from genomic DNA of *P. taiwanensis* VLB120. The three fragments were assembled using the NEBuilder HiFi DNA Assembly Master Mix.
pEMG-PVLB_23545/40-P_{14e}-*tyrA*^{fbr}	The pEMG-PVLB_23545/40 backbone was PCR-amplified from pEMG-PVLB_23545/40_BG13 (to be published) with primers BW575/BW1009, P_{14e}*(BCD2)* from pBG17 with primers BW1010/BW1011, and *tyrA*^{fbr} from pBG42-*aroG*^{fbr}-*tyrA*^{fbr} with primers BW1012/BW524. The three fragments were assembled using the NEBuilder HiFi DNA Assembly Master Mix.
pEMG-PVLB_23545/40-P_{14e}-*tyrA*^{fbr}-*aroG*^{fbr}	The pEMG-PVLB_23545/40 backbone was PCR-amplified from pEMG-PVLB_23545/40_BG13 (to be published) with primers BW575/BW1009, P_{14e}*(BCD2)* from pBG17 with primers BW1010/BW1011, and *tyrA*^{fbr} and *aroG*^{fbr} from pBG42-*aroG*^{fbr}-*tyrA*^{fbr} with primers BW1012/BW1013 and BW1014/BW1015, respectively. The four fragments were assembled using the NEBuilder HiFi DNA Assembly Master Mix.
pBG14f_*FRT_Kan_HaTAL1*	The pBG14f_*FRT_Kan* backbone was PCR-amplified with primers BW572/BW575. *HaTAL1* was amplified from pJNN-*HaTAL1* (unpublished) with primers BW887/BW888. The plasmid was assembled using the NEBuilder HiFi DNA Assembly Master Mix.
pBG14f_*FRT_Kan_SeSam8*	The pBG14f_*FRT_Kan* backbone was PCR-amplified with primers BW572/BW575. *SeSam8* was amplified from pJNN-*SeSam8* (unpublished) with primres BW889/BW890. The plasmid was assembled using the NEBuilder HiFi DNA Assembly Master Mix.
pBG14f_*FRT_Kan_RsTAL*	The pBG14f_*FRT_Kan* backbone was PCR-amplified with primers BW572/BW575. *RsTAL* was amplified from pJNN-*RsTAL* (unpublished) with primers BW891/BW892. The plasmid was assembled using the NEBuilder HiFi DNA Assembly Master Mix.
pBG14f_*FRT_Kan_FjTAL*	The pBG14f_*FRT_Kan* backbone was PCR-amplified with primers BW572/BW575. *FjTAL* was amplified from pJNN-*FjTAL* (unpublished) with primers BW893/BW894. The plasmid was assembled using the NEBuilder HiFi DNA Assembly Master Mix.
pBG14f_*FRT_Kan_StsTAL*	The pBG14f_*FRT_Kan* backbone was PCR-amplified with primers BW572/BW575. *StsTAL* was synthesized with suitable overlaps. The plasmid was assembled using the NEBuilder HiFi DNA Assembly Master Mix.
pBG14f_*FRT_Kan_RtPAL*	The pBG14f_*FRT_Kan* backbone was PCR-amplified with primers BW572/BW575. *RtPAL* was amplified from pJNN-*RtPAL* (unpublished) with primers BW603/BW886. The plasmid was assembled using the NEBuilder HiFi DNA Assembly Master Mix.
pJNNopt-*FjTAL*	The pJNNopt backbone was PCR-amplified with primers BW897/BW898. *FjTAL* was amplified from pBG14f_*FRT_Kan-FjTAL* with primers BW893/BW1162. The plasmid was assembled using the NEBuilder HiFi DNA Assembly Master Mix.
pJNNopt-*StsTAL*	The pJNNopt backbone was PCR-amplified with primers BW897/BW898. *StsTAL* was amplified from pBG14f_*FRT_Kan-StsTAL* with primers BW1163/BW1164. The plasmid was assembled using the NEBuilder HiFi DNA Assembly Master Mix.
pJNNopt-*RtPAL*	The pJNNopt backbone was PCR-amplified with primers BW897/BW898. *RtPAL* was amplified from pJNT*palpdc* with primers BW899/BW900. The plasmid was assembled using the NEBuilder HiFi DNA Assembly Master Mix.
pBG14f_*FRT_Kan_RtPAL-pdc*	The pBG14f_*FRT_Kan_RtPAL* fragment was PCR-amplified with primers BW938/BW939. *pdc* was amplified from pJNT*palpdc* with primers BW940/BW941. The plasmid was assembled using the NEBuilder HiFi DNA Assembly Master Mix.
pBG14f_*FRT_Kan_RtPAL-pdc-styABC*	The pBG14f_*FRT_Kan_RtPAL-pdc* fragment was PCR-amplified with primers BW938/BW659. *styABC* was amplified from pJNNopt-*RtPAL-pdc-styABC* with primers BW1189/BW1262. The plasmid was assembled using the NEBuilder HiFi DNA Assembly Master Mix.
pJNT*palpdc-styABC*	pJNT*palpdc* was digested with NheI. *styABC* was PCR-amplified with BW1226/BW1227 from pJNNopt-*RtPAL-pdc-styABC*. The plasmid was assembled using the NEBuilder HiFi DNA Assembly Master Mix.
pJNNopt-*RtPAL-pdc-styABC*	The pJNNopt-*RtPAL-pdc* fragment was PCR-amplified with primers BW897/BW659. *styAB* and *styC* were amplified from the megaplasmid pSTY with BW1189/BW1184 and BW1185/1190, repectively. The plasmid was assembled using the NEBuilder HiFi DNA Assembly Master Mix.
pBG14f_*FRT_Kan_RtPAL-ech-vdh-fcs*	The pBG14f_*FRT_Kan_RtPAL* fragment was PCR-amplified with primers BW938/BW939. The fragment *ech-vdh-fcs* was amplified from genomic DNA of *P. putida* KT2440 with primers BW944/BW945. The plasmid was assembled using the NEBuilder HiFi DNA Assembly Master Mix.

Table S3 List of cloning PCR primers. Shown are their respective designations, sequence, and description. Lower-case letters indicate overhangs, upper-case letters represent the binding sequence.

Name	Sequence (5' → 3')	Description
BW524	cgggtaccgagctcgaattcTTACTGGCGGTTGTCGTTGGCTTGA	*tyrA*^{fbr} reverse primer
BW539	gggtaatctgaattcgagctcAACGCCCTGGGCCTGGAG	TS1-*pheA*^{P144S} forward primer
BW540	agttctccacgctCACCACGCCGAAGTTGACG	TS1-*pheA*^{P144S} reverse primer
BW541	cggcgtggtgagcGTGGAGAACTCCACCGAAGG	TS2-*pheA*^{P144S} forward primer
BW542	gaagcttgcatgcctgcaggtcgacCGGGTCAGGTCGATGCCG	TS2-*pheA*^{P144S} reverse primer
BW572	TAGAAAACCTCCTTAGCATG	pBG14f_*FRT_Kan* reverse primer
BW575	GAATTCGAGCTCGGTACC	pBG14f_*FRT_Kan* and pEMG-PVLB_23545/40_P$_{em7}$ reverse primer
BW603	catgctaaggaggttttctaATGGCACCCTCGCTCGAC	*RtPAL* forward primer
BW659	TTACTTATTTAAACGATGGTAGTTTTG	pJNNopt-*RtPAL-pdc* reverse primer
BW886	cgggtaccgagctcgaattcCTAAGCGAGCATCTTGAGGAGG	*RtPAL* reverse primer
BW887	catgctaaggaggttttctaATGAGCACCACCCTGATTC	*HaTAL1* forward primer
BW887	cgggtaccgagctcgaattcTTAGCGAAACAGAATAATACTACGC	*HaTAL1* reverse primer
BW889	catgctaaggaggttttctaATGACCCAGGTTGTTGAAC	*SeSam8* forward primer
BW890	cgggtaccgagctcgaattcTTAGCCAAAATCTTTACCATCTG	*SeSam8* reverse primer
BW891	catgctaaggaggttttctaATGCTGGCTATGAGTCCTC	*RsTAL* forward primer
BW892	cgggtaccgagctcgaattcTTAAACTGGACTCTGTTGC	*RsTAL* reverse primer
BW893	catgctaaggaggttttctaATGAACACCATCAACGAG	*FjTAL* forward primer
BW894	cgggtaccgagctcgaattcTTAGTTGTTAATCAGGTGG	*FjTAL* reverse primer
BW897	TAGAGGGACAAACTCAAG	pJNNopt forward primer
BW898	TAGAAAACCTCCTTAGCATG	pJNNopt reverse primer
BW899	catgctaaggaggttttctaATGGCACCCTCGCTCGAC	*RtPAL* forward primer
BW900	accttgagtttgtccctctaCTAAGCGAGCATCTTGAGGAGG	*RtPAL* forward primer
BW938	GGGGATCCTCTAGAGTCG	pBG14f_*FRT_Kan_RtPAL* and pJNNopt-*RtPAL-pdc* forward primer
BW939	GGGTACCGAGCTCGAATTC	pBG14f_*FRT_Kan_RtPAL* reverse primer
BW940	ggaattcgagctcggtaccctcagaggaggtaccaacATGACAAAAACTTTTAAAACACTTG	*pdc* forward primer
BW941	gtcgactctagaggatccccTTACTTATTTAAACGATGGTAGTTTTG	*pdc* reverse primer
BW944	ggaattcgagctcggtacccgatttaggaggttgatctATGAGCAAATACGAAGGCCGC	*ech-vdh-fcs* forward primer
BW945	gtcgactctagaggatccccTCAAGGCCGCACCTTGGC	*ech-vdh-fcs* reverse primer
BW1009	TTAATTAAAGCGCTGATCGC	pEMG-PVLB_23545/40_BG13 forward primer
BW1010	gcgatcagcgctttaattaaCGGAGTTGACAACACTCG	P_{14e}*(BCD2)* forward primer
BW1011	ctgcaaccatTAGAAAACCTCCTTAGCATG	P_{14e}*(BCD2)* reverse primer
BW1012	aggttttctaATGGTTGCAGAACTGACC	*tyrA*^{fbr} forward primer
BW1013	ataactacctccttaggccggagcagggactagggTTACTGGCGGTTGTCGTTG	*tyrA*^{fbr} reverse primer
BW1014	ccctagtccctgctccggcctaaggaggtagttatATGAACTACCAAAACGATGATCTGCGCATCAAGG	*aroG*^{fbr} forward primer
BW1015	cgggtaccgagctcgaattcTTAGCCGCGCCGGGCCTT	*aroG*^{fbr} reverse primer
BW1162	accttgagtttgtccctctaTTAGTTGTTAATCAGGTGG	*FjTAL* reverse primer
BW1163	catgctaaggaggttttctaATGCCGAGCCTGGACTCC	*StsTAL* forward primer
BW1164	accttgagtttgtccctctaTTAGGCCGCACCCGTCAA	*StsTAL* reverse primer
BW1180	accttgagtttgtccctctaTTACTTATTTAAACGATGGTAGTTTTGATCAAAGTACTTGC	*RtPAL-pdc* reverse primer
BW1184	gcctcaccggCGTGCGCAATCAATTCAG	*styAB* reverse primer
BW1185	attgcgcacgCCGGTGAGGCCTTTCTGTG	*styC* forward primer
BW1189	accatcgtttaaataagtaacttaaagtaggaggtcatagagATGAAAAAGCGTATCGGTATTG	*styAB* and *styABC* forward primer
BW1190	accttgagtttgtccctctaTCATTCCGCAGCAGCGTG	*styC* reverse primer
BW1226	ccatcgtttaaataagtaagCTTAAAGTAGGAGGTCATAGAGATGAAAAAG	*styABC* forward primer
BW1227	ctcggcgcggcgggtcagtgTCATTCCGCAGCAGCGTG	*styABC* reverse primer
BW1262	gtcgactctagaggatccccTCATTCCGCAGCAGCGTG	*styABC* reverse primer

Table S4 Sequences of genes that were codon-optimized for *P. taiwanensis* VLB120.

FjTAL (5' → 3') (Lenzen et al., 2019)

ATGAACACCATCAACGAGTATCTGTCGTTGGAGGAGTTCGAGGCCATTATTTTCGGGAACCAAAAGGTCACCATCAGCGACGTCGT
CGTCAATCGCGTCAACGAATCCTTCAACTTTCTGAAGGAGTTCAGCGGCAACAAGGTGATCTACGGCGTGAACACCGGCTTTGGCC
CGATGGCGCAGTACCGTATCAAGGAGAGCGATCAAATCCAGCTGCAATACAACCTGATCCGCAGCCATAGCTCGGGGACCGGGAAG
CCGCTGTCCCCGGTCTGTGCCAAGGCCGCTATCTTGGCCCGCCTGAACACCCTGAGCCTCGGGAACAGCGGCGTGCACCCTAGCGT
AATTAATTTGATGAGCGAACTGATCAACAAGGACATCACGCCGCTGATCTTCGAACACGGCGGGGTGGGCGCGAGTGGCGACCTGG
TACAGCTGTCGCACTTGGCGCTGGTCCTGATCGGCGAAGGTGAAGTCTTCTACAAAGGCGAGCGCCGTCCGACCCCGGAGGTGTTT
GAAATCGAGGGCCTCAAGCCAATCCAGGTCGAGATCCGCGAAGGCCTGGCCTTGATCAACGGTACGAGCGTGATGACCGGCATCGG
TGTCGTGAACGTATATCATGCGAAAAAGTTGCTGGACTGGTCGCTGAAGTCCAGCTGCGCCATCAACGAACTGGTCCAAGCCTACG
ACGATCACTTCAGCGCCGAACTGAACCAGACGAAACGCCACAAAGGCCAACAGGAGATCGCGCTGAAGATGCGTCAGAACCTCAGC
GATTCCACCTTGATCCGGAAGCGCGAGGACCACCTGTACTCCGGCGAAAACACCGAGGAAATCTTCAAGGAAAAAGTTCAGGAGTA
CTACAGCCTGCGCTGTGTGCCGCAAATCCTGGGTCCTGTGCTGGAGACGATCAACAACGTTGCCTCGATCCTCGAAGATGAGTTTA
ACAGCGCGAATGATAACCCGATTATCGACGTCAAGAACCAACACGTCTACCACGGCGGCAACTTCCACGGCGACTACATTTCCCTG
GAAATGGACAAACTGAAGATCGTGATCACCAAGCTGACGATGCTGGCCGAACGCCAACTGAATTACTTGCTGAACAGCAAGATCAA
TGAGCTGCTGCCCCCTTTTGTCAACCTGGGTACGCTGGGTTTCAACTTCGGTATGCAAGGGGTGCAATTTACCGCCACCAGCACCA
CCGCGGAATCGCAGATGCTGAGTAACCCGATGTACGTTCACTCCATTCCGAACAACAACGACAATCAGGACATTGTGAGCATGGGC
ACGAACTCCGCGGTAATCACCTCCAAAGTGATCGAAAACGCCTTCGAGGTACTGGCCATTGAAATGATCACCATTGTTCAGGCGAT
TGACTACCTGGGTCAGAAGGACAAGATCAGCTCGGTGTCGAAGAAGTGGTACGATGAAATCCGCAACATCATCCCTACGTTCAAAG
AAGACCAAGTTATGTATCCTTTCGTGCAGAAGGTGAAAGACCACCTGATTAACAACTAA

StsTAL (5' → 3') (This study)

ATGCCGAGCCTGGACTCCATCGTTGAGGCCGCGAGCTGGACTGCCAAGTTGGGCCCCCTCACTGACGCGGACGTCGCTCGCATGGA
TCGCTCGGGGGCCACCGTTGATGCCTACCTGGCTGAGGGTCGTCCTGTATATGGTCTGACGCAGGGCTTCGGCCCGCTGGTTACCT
ATAGCGCTACCTCGGAGATGGAGCAAGGCGCGAGCCTGATCAGCCATCTGGGCACTGCGCAGGGGCGTCCTATCGACCCCGATGCG
TCGCGCCTGGTCTTCTGGCTGCGCCTCAACAGTATGCGTAAGGGCTTCAGCGCAGTCTCGACCGAGTTTTGGCAACGTCTGGCTGA
CCTGTGGAACGCCGGCTTTACTCCTGTAATCCCCCGCGACGGCACTGTGAGTGCAAGCGGTGACTTGCAGCCCTTGGCTCACGTGG
CGCTGGCCTGCGCCGGTCATGGCGAAGCCTGGGTGCGCGATGAACAGGATCGTTGGACCCGTCGCCCAGCAGCTGAAGCACTGGCT
GGTCTGGGTGCTGAACCGCTGGTGTGGCCCGTCCGCGAGGCGCTGGCATTCGTAAACGGCACCGGTGTAGGCTTGGCCGTCGCCAT
CTTGAACCAGCGCTCCGCTGTGCGTCTGGTGCGTGCTGTGGCGACTCTGACCGCACGTTTGACCGACCTGTTGGGCGGCAATGCCG
AACACTACGATGAAGGTGTGGGTCAAGCCCGTAATCAGCTGGGCCAGTTGGAAGTAGCGCGCTGGATCCGCGCCGAAATCCCTGCC
GGTCATCGGCGTGATGAGCGTCGGCCCCTGCAAGAGCCGTATAGTCTGCGCTGCGCCCCGCAGGTACTGGGCGCAGTCCTGGACCA
ACTGACCACTGCCGGTGAGATCCTCCTGCGCGAGGCCAACGGTTGTACCGACAATCCCTTGACCTACGAGGACCGCGTTCTCCACG
CGGGTAACTTCCATGCCATGCCCGTTGGCTTCGCGAGCGAGCAGACGGGGCTGGCCATGCACATGGCCGCGTACCTCGCCGAACGt
CAGTTGGGGCTGGTGGTGAATCCGACGACCAACGGCGACCTGCCGATCATGCTGACCCCACGCGCTGGGCGTGGTTGTGGCCTGGC
TGGTGTACAAATTAGCGCGACCAGCTTTATCAGTCGCATCCGCCAACTGGTGACCCCGGCCTCGCTGACCACCCTCCCGACGAACG
GCTGGAACCAGGACCATGTGCCAATGGCTCTCAATGGTGCAAACGGCGTCGGCGAAGCGTTGGAGCTGGGCTGGTTGGCAGTAGGT
AGTCTGGCCTTGGCGGCTGCCCAATTGGCCGTCATGACTGGCAAAGCTGAGAGTGCCACCGGTGTCTGGGCGGAGCTGGCCCGCAT
TAGCCCGGCACTCGACGCAGACCGCCCCATGGCTGGCGAAGTCCGTGCCGCTGCGGAACTGTTCCGCGATCACGCTGAACGCCAGT
TGACGGGTGCGGCCTAA

tyrA^{fbr} (5' → 3') (Wynands et al., 2018)

ATGGTTGCAGAACTGACCGCGCTGCGTGACCAAATCGACGAGGTGGACAAAGCACTGTTGAACCTCCTGGCTAAGCGCCTGGAGCT
GGTGGCCGAGGTGGGCGAGGTCAAAAGTCGCTTTGGCCTGCCTATTTACGTTCCTGAGCGCGAGGCAAGCATCCTGGCAAGCCGTC
GCGCCGAGGCTGAGGCCCTGGGTGTGCCCCCCGACTTGATCGAGGATGTCCTGCGCCGCGTGATGCGTGAAAGCTACAGCTCGGAG
AACGACAAGGGCTTCAAGACCTTGTGCCCCTCCTTGCGCCCAGTCGTGATTGTCGGCGGGGGGGCCAGATGGGCCGTTTGTTCGA
AAAGATGCTCACGCTGAGCGGCTACCAGGTGCGCATCCTGGAACAGCACGACTGGGACCGGGCGGCCGACATCGTCGCTGATGCCG
GTATGGTAATCGTAAGCGTCCCCATCCATGTGACCGAACAGGTGATTGGCAAGCTGCCTCCGTTGCCGAAGGACTGCATCCTGGTG
GATCTCGCGAGCGTGAAGAACGGCCCGCTGCAAGCGATGCTGGTTGCCCATGATGGCCCGGTGCTGGGCCTGCACCCAATGTTCGG
CCCAGACAGCGGCTCGCTGGCAAAGCAAGTCGTGGTGTGGTGCGACGGTCGCAAACCCGAGGCGTATCAATGGTTCTTGGAACAGA
TCCAGGTTTGGGGTGCCCGTTTGCACCGCATCTCCGCAGTCGAGCATGACCAGAATATGGCTTTCATCCAGGCCCTGCGCCATTTC
GCCACCTTCGCGTACGGCCTGCACCTGGCAGAGGAGAACGTGCAGCTGGAGCAGCTGCTGGCACTCAGCTCCCCCATCTACCGCCT
GGAGCTGGCCATGGTCGGTCGCCTGTTCGCCCAGGACCCGCAGCTGTACGCGGACATCATCATGTCGTCCGAACGCAATCTCGCGC
TGATCAAGCGCTATTATAAACGCTTCGGCGAGGCGATCGAACTGCTGGAACAGGGCGATAAGCAAGCCTTCATCGACTCGTTCCGC
AAGGTGGAACATTGGTTCGGCGACTATGTCCAGCGCTTCCAGTCGGAGTCGCGCGTTCTGTTGCGTCAAGCCAACGACAACCGCCA
GTAA

aroG^{fbr} (5' → 3') (Wynands et al., 2018)

ATGAACTACCAAAACGATGATCTGCGCATCAAGGAAATCAAAGAACTCCTGCCTCCCGTAGCACTCCTGGAGAAGTTCCCAGCGAC
CGAGAACGCCGCCAATACCGTGGCCCACGCCCGTAAGGCCATCCACAAGATCCTCAAAGGCAACGACGATCGCTTGTTGGTCGTCA
TCGGGCCGTGCTCGATCCACGACCCGGTCGCGGCCAAGGAGTACGCGACCCGCCTGCTGGCGCTCCGCGAAGAGCTGAAAGACGAA
CTGGAAATCGTCATGCGCGTGTATTTCGAAAAGCCACGGACTACTGTGGGCTGGAAGGGTTTGATCAACGATCCTCATATGGACAA
CAGCTTCCAGATTAACGACGGTCTCCGCATCGCCCGCAAATTGCTGCTCGATATCAACGACAGCGGCCTGCCAGCAGCCGGCGAGT
TCCTCAATATGATCACCCCCCAGTATCTCGCAGACCTGATGAGCTGGGGCGCTATTGGCGCGCGGACCACCGAGTCGCAAGTACAC
CGGGAACTGGCTTCGGGCCTGAGCTGTCCTGTCGGCTTCAAGAACGGGACCGACGGCACCATCAAGGTGGCGATCGACGCCATCAA
CGCGGCGGGCGCCCCACACTGCTTCTTGAGTGTCACCAAGTGGGGCCACAGTGCGATTGTCAATACCTCCGGCAACGGGGACTGCC
ACATTATCCTGCGGGGCGGCAAGGAACCCAACTACAGCGCGAAGCACGTGGCCGAGGTTAAAGAGGGCCTCAACAAGGCTGGGCTG
CCGGCCCAGGTGATGATCGACTTCAGCCATGCGAATAGCTCCAAGCAGTTCAAGAAACAGATGGATGTTTGTGCCGATGTGTGTCA
GCAGATCGCCGGTGGCGAAAAAGCCATCATCGGCGTTATGGTGGAGAGCCACCTCGTCGAGGGCAACCAGTCGCTGGAAAGCGGCG
AGCCTCTGGCTTACGGTAAAAGCATCACCGACGCCTGCATCGGCTGGGAGGACACCGACGCCCTGCTGCGCCAGCTGGCGAATGCC
GTGAAGGCCCGGCGCGGCTAA

Table S5 Elution profile used for the InfinityLab Poroshell 120 EC-C18.

time (min)	0.1% (v/v) TFA (%)	acetonitrile (%)
00.00	95	5
02.00	95	5
10.00	30	70
12.00	30	70
13.00	95	5
14.00	95	5

Table S6 Elution profile used for the ISAspher 100-5 C18 BDS.

time (min)	0.1% (v/v) TFA (%)	acetonitrile (%)
00.00	90	10
04.00	90	10
12.00	5	95
16.00	5	95
18.00	90	10
20.00	90	10

References

Ackermann, Y. S., Li, W. J., Op de Hipt, L., Niehoff, P. J., Casey, W., Polen, T., Köbbing, S., Ballerstedt, H., Wynands, B., O'Connor, K., Blank, L. M., Wierckx, N., 2021. Engineering adipic acid metabolism in *Pseudomonas putida*. Metabolic engineering. 67, 29-40.

Choi, K. H., Gaynor, J. B., White, K. G., Lopez, C., Bosio, C. M., Karkhoff-Schweizer, R. R., Schweizer, H. P., 2005. A Tn7-based broad-range bacterial cloning and expression system. Nature methods. 2, 443-448.

Figurski, D. H., Helinski, D. R., 1979. Replication of an origin-containing derivative of plasmid RK2 dependent on a plasmid function provided *in trans*. Proc Natl Acad Sci U S A. 76, 1648-1652.

Lenzen, C., Wynands, B., Otto, M., Bolzenius, J., Mennicken, P., Blank, L. M., Wierckx, N., 2019. High-yield production of 4-hydroxybenzoate from glucose or glycerol by an engineered *Pseudomonas taiwanensis* VLB120. Frontiers in bioengineering and biotechnology. 7, 130.

Martínez-García, E., de Lorenzo, V., 2011. Engineering multiple genomic deletions in Gram-negative bacteria: analysis of the multi-resistant antibiotic profile of *Pseudomonas putida* KT2440. Environ Microbiol. 13, 2702-2716.

Neves, D., Vos, S., Blank, L. M., Ebert, B. E., 2019. *Pseudomonas* mRNA 2.0: Boosting gene expression through enhanced mRNA stability and translational efficiency. Frontiers in bioengineering and biotechnology. 7, 458.

Verhoef, S., Wierckx, N., Westerhof, R. G., de Winde, J. H., Ruijssenaars, H. J., 2009. Bioproduction of *p*-hydroxystyrene from glucose by the solvent-tolerant bacterium *Pseudomonas putida* S12 in a two-phase water-decanol fermentation. Applied and environmental microbiology. 75, 931-936.

Wynands, B., Lenzen, C., Otto, M., Koch, F., Blank, L. M., Wierckx, N., 2018. Metabolic engineering of *Pseudomonas taiwanensis* VLB120 with minimal genomic modifications for high-yield phenol production. Metabolic engineering. 47, 121-133.
